# Enhanced preoperative prediction for microvascular invasion in hepatocellular carcinoma through an optimized MR Radiomics combination strategy and machine learning predictor

**DOI:** 10.3389/fmed.2026.1764733

**Published:** 2026-02-11

**Authors:** Mengting Feng, Yingjian Yang, Zongbo Dai, Ziran Chen, Longyu Li, Zewei Wu, Xuejian Li, Tingwei Guo, Yiman Meng, Qiang Li, Zihao Zhao, Tao Li, Jialin Zhang, Yan Kang

**Affiliations:** 1 College of Medicine and Biological Information Engineering, Northeastern University, Shenyang, China; 2 Department of Radiological Research and Development, Shenzhen Lanmage Medical Technology Co., Ltd., Shenzhen, Guangdong, China; 3 Department of Hepatobiliary Surgery, The First Hospital of China Medical University, Shenyang, China; 4 College of Health Science and Environmental Engineering, Shenzhen Technology University, Shenzhen, China; 5 School of Data and Computer Science, Shandong Women’s University, Jinan, Shandong, China; 6 College of Applied Sciences, Shenzhen University, Shenzhen, Guangdong, China; 7 Faculty of Data Science, City University of Macau, Macao, Macao SAR, China

**Keywords:** enhanced T1-weighted magnetic resonance imaging, hepatocellular carcinoma, machine learning algorithms, microvascular invasion, preoperative prediction, radiomics

## Abstract

**Background:**

Preoperative prediction of microvascular invasion (MVI) in hepatocellular carcinoma (HCC) is a crucial step toward personalized treatment, improved treatment outcomes, and enhanced patient survival. However, the disadvantage of existing prediction models of MVI in HCC based on enhanced magnetic resonance imaging (MRI) is that they require combining non-imaging information from enhanced MRI, or determining the perioperative region is highly subjective. These disadvantages are not conducive to the clinical application of predictive models, which hinders clinical decision-making and management for these vulnerable populations.

**Methods:**

To address the problem of combining non-imaging information from enhanced MRI with the highly subjective determination of the perioperative region, we propose an enhanced preoperative prediction model for MVI in HCC using an optimized MR Radiomics combination strategy and a machine learning predictor. First, the HCC was manually segmented from 125 × 512 × 512 × *N* abdominal enhanced T1-weighted magnetic resonance imaging (T1WI) images during the arterial phase, generating 125 × 512 × 512 × *N* HCC mask images. Second, 125 × 1,692 MR Radiomics features of HCC are extracted from abdominal enhanced T1WI images based on the HCC mask images. Third, the 125 × *N* selected and 125 × 10 fused MR Radiomics features are determined using the proposed optimized MR Radiomics combination strategy with 5-fold cross-validation. Finally, the best preoperative prediction model is constructed using a random forest (RF) predictor with 125 × *N* selected and 125 × 10 fused MR Radiomics features.

**Results:**

The proposed MVI preoperative prediction model (RF + LASSO + SPECTRAL-10) achieves a mean accuracy of 0.7520 ± 0.0867, a mean precision of 0.7354 ± 0.1863, a mean recall of 0.6955 ± 0.2203, a mean *F*_1_-score of 0.6943 ± 0.1437, and a mean AUC of 0.7962 ± 0.1700.

**Discussion:**

The proposed best preoperative prediction model can effectively predict MVI in HCC, potentially serving as a strong decision-making tool for these vulnerable populations.

## 1 Introduction

Hepatocellular carcinoma (HCC) has become the most common type of primary liver cancer, accounting for approximately 85–90% of all cases, resulting in the fourth leading cause of cancer-related deaths globally, with China accounting for 42% of new cases annually in 2022 and a 5-year survival rate of only 18% for advanced patients (1–3). Microvascular invasion (MVI) is a significant risk factor for postoperative recurrence and metastasis of HCC (4).

Preoperative prediction of MVI in HCC is a crucial step toward personalized treatment, improved treatment outcomes, and enhanced patient survival (4–7). It also plays an irreplaceable role in optimizing clinical decision-making and improving patient prognosis (8). First, for MVI-positive patients, preoperative prediction can prompt doctors to expand the surgical margin and use a wider resection range (e.g., wide-margin resection) to reduce the risk of tumor cell residue and recurrence (9, 10). Conversely, for MVI-negative patients, the surgical margin can be appropriately reduced to minimize surgical trauma. Second, MVI status can help determine whether more precise surgical methods, such as anatomical liver resection, are needed (10, 11). For MVI-positive patients, anatomical liver resection may be more beneficial for thoroughly clearing the tumor and possible micro-metastases in the surrounding area. Third, patients with a high risk of MVI predicted before surgery may be more suitable for neoadjuvant therapy, such as chemotherapy and targeted therapy, to reduce tumor volume, decrease the degree of MVI, improve the surgical resection rate, and achieve a curative effect (12). Fourth, postoperative adjuvant treatment plans can be formulated in advance based on the preoperative MVI prediction results (13). For MVI-positive or high-risk patients, more aggressive adjuvant therapy, such as hepatic artery infusion chemotherapy and targeted therapy, may be needed after surgery to further reduce the risk of recurrence. In addition, for MVI-negative patients, the intensity and duration of adjuvant therapy can be adjusted appropriately. Finally, MVI is an essential predictor of postoperative recurrence and metastasis in hepatocellular carcinoma (4). Preoperative prediction of MVI status can help doctors more accurately evaluate patient prognosis and provide more reasonable treatment recommendations and follow-up plans. For example, patients with positive MVI have a higher risk of postoperative recurrence and require closer follow-up and monitoring.

Compared with abdominal ultrasound imaging and computed tomography (CT), enhanced T1-weighted magnetic resonance imaging (T1WI) is considered the preferred imaging modality for preoperative prediction of MVI in HCC. Compared with abdominal ultrasound, CT and magnetic resonance imaging (MRI) offer higher spatial resolution and stronger lesion guidance for deep tumors, such as HCC in the liver (14). Compared with abdominal CT imaging, MRI also offers high soft-tissue resolution and is superior to CT and ultrasound in detecting small lesions in solid organs, such as the liver (e.g., liver cancer ≤1 cm) (15). It can also identify tumor boundaries and adjacent nerves and blood vessels, helping detect early liver cancer and determine the relationship between tumors and surrounding tissues. Meanwhile, MRI avoids X-ray exposure and is suitable for children, pregnant women, and patients who require multiple examinations, especially for liver cancer patients who require long-term follow-up, which is safer (16, 17). Compared with enhanced CT, enhanced T1WI can effectively display the anatomical structures of liver organs, and this comparison helps doctors observe their morphology and position (18). Meanwhile, liver tumor lesions, such as HCC, also exhibit different signal characteristics on enhanced T1WI images due to changes in tissue characteristics, indicating that the tumor may be invasive and invade surrounding liver tissue. Therefore, T1WI is an essential sequence in MRI of HCC, providing key information for diagnosis, staging, treatment plan selection, and efficacy evaluation. Finally, selecting abdominal T1WI images acquired during the late arterial phase is crucial for preoperative prediction of MVI in HCC. Specifically, during the arterial phase, mainly the late arterial phase, of dynamic enhanced MRI, liver tumors show uniform or uneven significant enhancement. In contrast, their enhancement in the portal vein phase and/or delayed phase is lower than that of the liver parenchyma (19–21). Therefore, the arterial phase of liver tumors shows significant enhancement, while the portal vein phase shows less enhancement than the liver parenchyma.

Currently, the two major mainstream approaches to prediction tasks are machine learning and deep learning (22–39). However, deep learning-based prediction tasks are suitable for scenarios with large amount of data, complex problems, and high performance requirements (22–24). It often requires more training data during the training process of the prediction network so that the prediction model can achieve a specific performance. Although some scholars have developed transfer learning strategies to overcome the aforementioned technical issues, this remains an insurmountable problem in medical imaging, where training data are scarce. Unlike deep learning, machine learning algorithms have advantages in handling small data sets and in computational resource requirements, making them suitable for scenarios with limited data or computational resources (37–39).

Radiomics, combined with machine learning algorithms, a technique for extracting and analyzing high-throughput quantitative features from medical images, has been widely used for auxiliary diagnosis, treatment, and prognosis (24–36) and has also opened new avenues for non-invasive preoperative prediction of MVI in HCC based on enhanced MRI (37–39). However, the disadvantage of existing prediction models of MVI in HCC based on enhanced MRI is that they require combining non-imaging information from enhanced MRI, such as aminotransferase-to-platelet ratio and gamma-glutamyl transferase-to-platelet ratio (22, 23, 38), or that determining the perioperative region is highly subjective (39). These disadvantages are not conducive to the clinical application of predictive models. Radiomics derived from abdominal enhanced T1WI images during the arterial phase should provide more information to enhance preoperative prediction of MVI in HCC. Therefore, it is necessary to propose a preoperative prediction model for MVI in HCC to address the challenge of combining non-imaging information from enhanced MRI with highly subjective perioperative region delineation. Our contributions in this study are briefly described as follows:

1. We propose a preoperative prediction model for MVI in HCC to eliminate non-imaging information of enhanced MRI and the highly subjective determination of perioperative region, which is conducive to the clinical application of the predictive model.
2. The linear and non-linear features extracted from the MR Radiomics features of HCC are fully exploited by the feature selection and feature fusion algorithms to adapt to different machine learning predictors.
3. The best preoperative prediction model is determined by the proposed optimized MR Radiomics combination strategy to seek the optimal combination of features.
4. The proposed best preoperative prediction model can effectively predict the MVI in HCC, which may become a strong decision-making tool for these vulnerable populations.

## 2 Materials and methods

This study proposes an enhanced preoperative prediction model for MVI in HCC using an optimized MR Radiomics combination strategy and a machine learning predictor. Based on the above, materials and methods are described in Sections 2.1 and 2.2, respectively.

### 2.1 Materials

**Figure 1:**
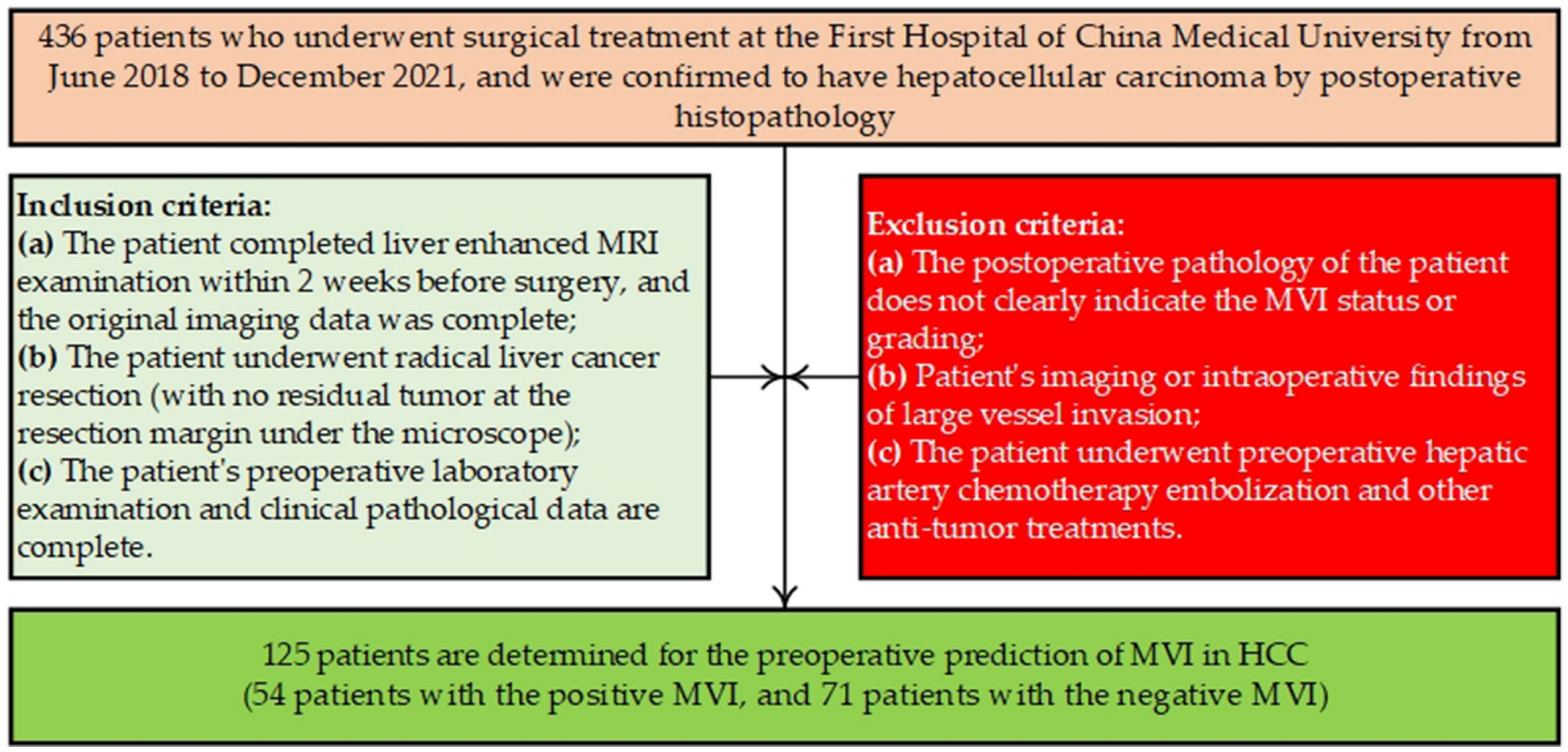
Flow diagram for patient selection in this study.

Figure 1 shows the flow diagram for patient selection in this study. Table 1 reports the characteristics of the dataset of the 125 patients. Specifically, all 125 patients underwent abdominal enhanced MRI scanning using the same 3.0 T MRI scanner (GE MEDICAL SYSTEMS, SIGNA Pioneer, Scanning Sequence: GR, Sequence Variant: SS/SK) before surgery. The contrast agent for enhanced MRI scanning is gadopentetamide (Gd-DTPA, Manufacturer: Beijing Beilu Pharmaceutical), which is injected intravenously at a dose of 0.2 mL/kg at a flow rate of 1.0 mL/s and then flushed with 20 mL of physiological saline. Abdominal enhanced MR images at T1-weighted imaging were collected at 30 s (arterial phase), 60 s (portal phase), and 120 s (delayed phase) after injection. After the surgery, two pathologists with ≥10 years of experience double-blindly reviewed the patients’ pathological sections to evaluate the presence of MVI.

**Table 1:** Characteristics of the dataset of the 125 patients.

MVI status	Characteristics	Value/mean ± SD^a^ (maximum to minimum)
Positive MVI	Case	54
	Sex (male/female)	43/11
	Age (years)	58.06 ± 9.52 (73 to 33)
	Flip angle (°)	15
	Pixel spacing	0.76 ± 0.03 (0.86 to 0.74)
	Slice thickness (mm)	5.00 ± 0.03 (5.20 to 5.00)
	Repetition time (ms)	3.05 ± 0.17 (3.47 to 2.83)
	Echo time (ms)	1.33 ± 0.17 (1.54 to 1.22)
	Spacing between slices (mm)	2.50 ± 0.01 (2.60 to 2.50)
	Percent phase field of view	80
	Pixel band width	473.21 ± 24.53 (488.28 to 434.02)
Negative MVI	Cases	71
	Sex (male/female)	58/13
	Age (years)	58.92 ± 8.24 (77 to 37)
	Flip angle (°)	15
	Pixel spacing	0.76 ± 0.02 (0.86 to 0.74)
	Slice thickness (mm)	5.02 ± 0.17 (6.00 to 4.60)
	Repetition time (ms)	3.02 ± 0.16 (3.38 to 2.82)
	Echo time (ms)	1.32 ± 0.09 (1.51 to 1.22)
	Spacing between slices (mm)	2.51 ± 0.09 (3.00 to 2.30)
	Percent phase field of view	80
	Pixel band width	476.05 ± 22.83 (488.28 to 434.02)

a SD, standard deviation.

Based on the above findings, 125 preoperative cases with enhanced abdominal T1WI images at the late arterial phase were retrospectively collected, and each case had an explicit postoperative MVI diagnosis (positive or negative MVI). The Medical Ethics Committee of the First Hospital of China Medical University approved this study.

### 2.2 Methods

**Figure 2:**
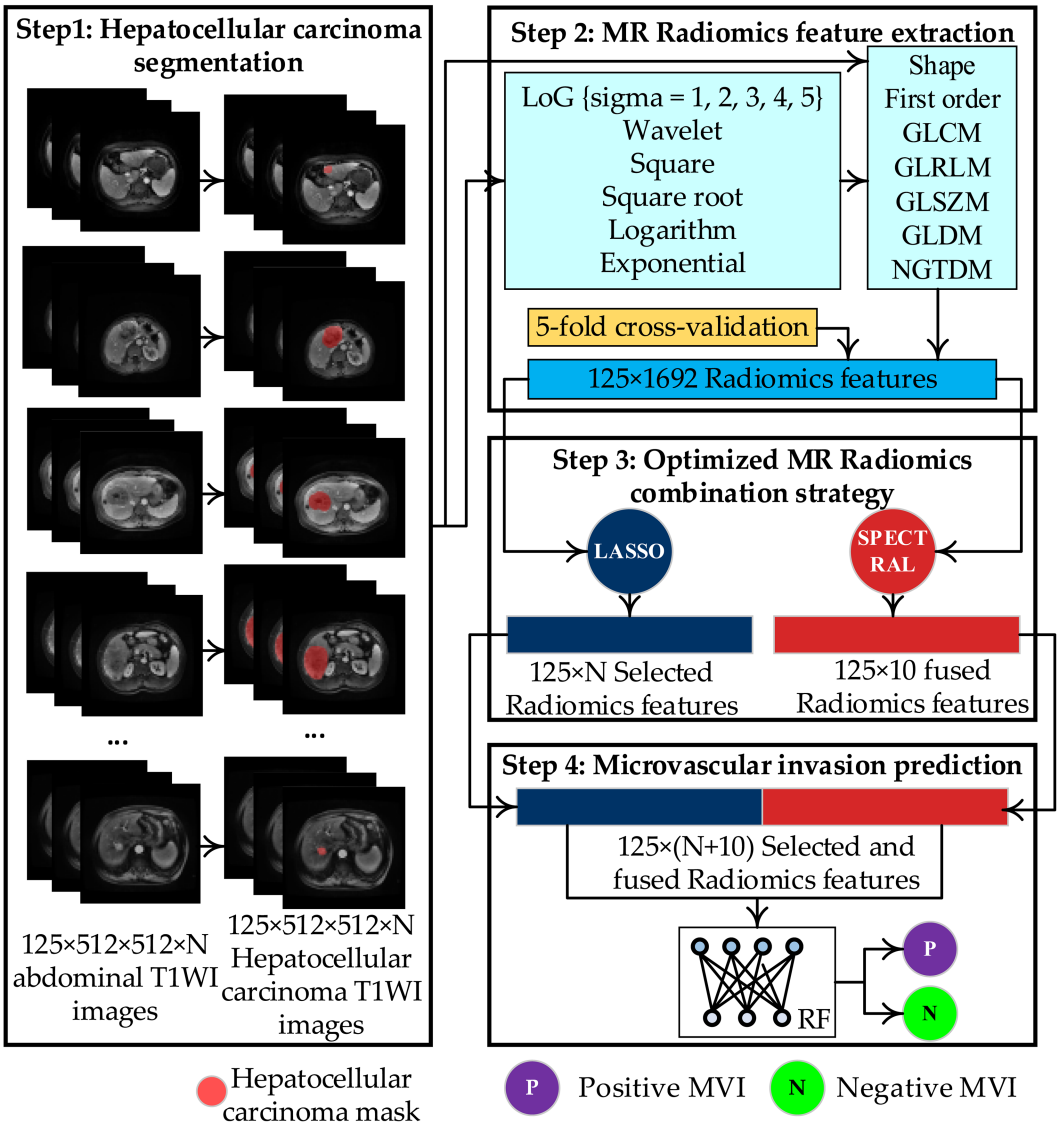
Construction process of the enhanced preoperative prediction model for microvascular invasion (MVI) in hepatocellular carcinoma (HCC) using an optimized MR Radiomics combination strategy and the random forest (RF) predictor.

Figure 2 shows the construction process of the enhanced preoperative prediction model for MVI in HCC using an optimized MR Radiomics combination strategy and the random forest (RF) predictor. First, the HCC was manually segmented from 125 × 512 × 512 × *N* abdominal enhanced T1WI images during the arterial phase, generating 125 × 512 × 512 × *N* HCC mask images. Second, 125 × 1,692 MR Radiomics features of HCC are extracted from abdominal enhanced T1WI images based on the HCC mask images. Third, the 125 × *N* selected and 125 × 10 fused MR Radiomics features are determined using the proposed optimized MR Radiomics combination strategy with 5-fold cross-validation. Finally, the best preoperative prediction model is constructed using the RF predictor with 125 × *N* selected and 125 × 10 fused MR Radiomics features.

#### 2.2.1 Step 1: HCC segmentation

The HCC was manually segmented from 125 × 512 × 512 × *N* abdominal enhanced T1WI images during the arterial phase, generating 125 × 512 × 512 × *N* HCC mask images. Specifically, two radiologists with ≥5 years of experience used ITK-SNAP software (v3.8.0, http://www.itksnap.org) on 125 × 512 × 512 × *N* abdominal enhanced T1WI images during the arterial phase to independently manually delineate the region of interest of the HCC and reach consensus through negotiation (intra-group correlation coefficient ICC >0.85). If there is no consensus through negotiation, then an experienced chief radiologist reviews and modifies the 125 × 512 × 512 × *N* HCC mask images.

#### 2.2.2 Step 2: MR Radiomics feature extraction

125 × 1,692 MR Radiomics features of HCC are extracted from abdominal enhanced T1WI images based on the HCC mask images. Specifically, PyRadiomics (24–36), a radiomics feature extraction model, is used to extract 125 × 1,692 MR Radiomics features of HCC from abdominal enhanced T1WI images using HCC mask images. First, six kinds of derived HCC images are generated using the Laplacian of Gaussian (LoG), wavelet, square, square root, logarithm, and exponential filters, respectively, based on the original HCC images. Then, 125 × 1,692 MR Radiomics features are extracted from the original and six derived HCC images based on the classes of radiomic features. The sigma values for LoG are 1.0, 2.0, 3.0, 4.0, and 5.0, and the wavelet filter includes the nine component combinations: LLH, LHL, LHH, HLL, HLH, HHL, HHH, and LLL. The classes of radiomic features include shape-based (2D and 3D) (shape), first-order statistics (first order), gray-level cooccurrence matrix (GLCM), gray-level run length matrix (GLRLM), gray-level size zone matrix (GLSZM), neighboring gray tone difference matrix (NGTDM), and gray-level dependence matrix (GLDM). These 125 × 1,692 MR Radiomics features of HCC are available in Supplementary Table S1.

#### 2.2.3 Step 3: Optimized MR Radiomics combination strategy

The 125 × *N* selected and 125 × 10 fused MR Radiomics features are determined using the proposed optimized MR Radiomics combination strategy and 5-fold cross-validation. Specifically, the proposed optimized MR Radiomics combination strategy consists of three key steps based on the 5-fold cross-validation. First, the least absolute shrinkage and selection operator (LASSO) (24–36, 38), a powerful method for variable selection and regularization in high-dimensional datasets with multicollinearity or a much larger number of variables than the sample size, is performed at each fold cross-validation (25 × 1,692 MR Radiomics features) to determine the 125 × *N* selected MR Radiomics features. Second, spectral embedding (SPECTRAL) (40, 41), a feature dimensionality reduction method based on graph theory, is performed at each fold cross-validation (the same 25 × 1,692 MR Radiomics features need to be selected by the LASSO) to determine the 125 × 10 fused MR Radiomics features. SPECTRAL constructs an adjacency graph or k-nearest neighbor graph based on the similarity between data points of MR Radiomics features in each fold of cross-validation, calculates the Laplacian matrix of the graph, and finally solves the eigenvectors of the matrix to obtain low-dimensional embeddings. Therefore, the core of SPECTRAL is to preserve the connectivity of data points in MR Radiomics features within the graph, which is very effective for discovering non-convex, streamlined cluster structures. Finally, the 25 × *N* selected and 25 × 10 fused MR Radiomics features at each fold cross-validation are combined to obtain the optimized MR Radiomics combination vector.

#### 2.2.4 Step 4: Microvascular invasion prediction

RF (26–36, 38), an ensemble learning method that improves a model’s generalization and robustness by combining predictions from multiple decision trees, is selected as the predictor for MVI in HCC. Specifically, sub-datasets from the optimized MR Radiomics combination vector are randomly selected with replacement, and different training samples are generated for each tree. Then, during node splitting in each decision tree, a portion of the MR Radiomics features in the sub-dataset is randomly selected to find the optimal splitting point and reduce feature correlation. Secondly, each tree grows independently on the sub-dataset until the stopping condition is met. Finally, the MVI status is predicted using majority voting.

## 3 Experiments and results

This section comprehensively implements the comparative experiment. Then, the preoperative prediction models based on different predictors and MR Radiomics features are presented.

### 3.1 Experiments

**Figure 3:**
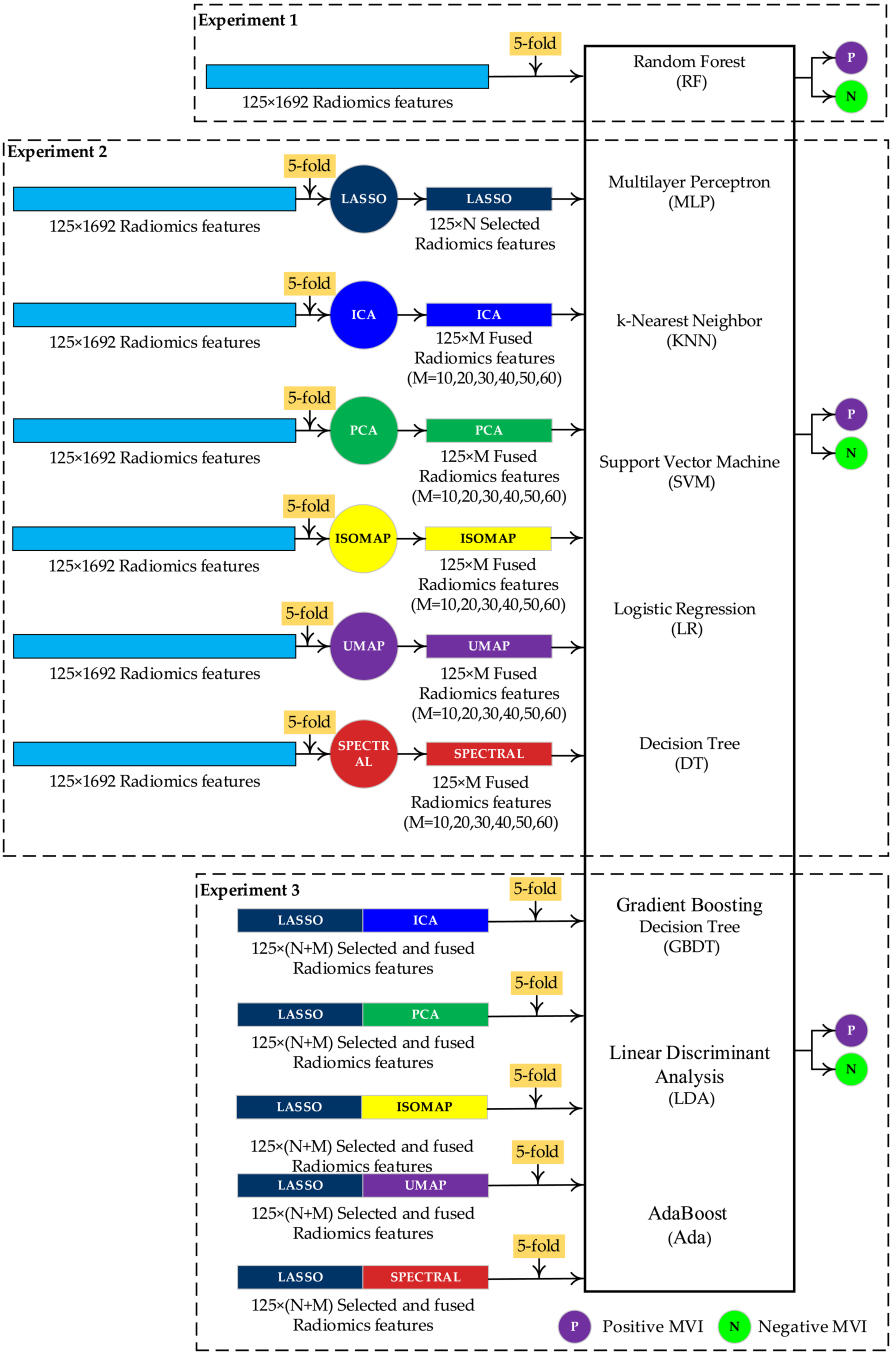
Experimental design for constructing and evaluating the microvascular invasion (MVI) preoperative prediction models.

Figure 3 shows the comparative experimental design for constructing and evaluating the MVI preoperative prediction models. Specifically, nine classic machine learning predictors (26–36, 38) are selected to perform the comparative experiment with different MR Radiomics features, including RF, multilayer perceptron (MLP), k-nearest neighbor (KNN), support vector machine (SVM), logistic regression (LR), decision tree (DT), gradient boosting decision tree (GBDT), linear discriminant analysis (LDA), and AdaBoost (Ada). Meanwhile, LASSO is selected as the feature selection algorithm (26–36). In addition, principal component analysis (PCA) (34), independent component analysis (ICA) (42), isometric mapping (ISOMAP) (43), uniform manifold approximation and projection (UMAP) (44), and SPECTRAL are selected as the feature fusion algorithms.

Experiment 1 is designed to evaluate preoperative prediction models based on the nine classic machine learning predictors and 125 × 1,692 Radiomics features using 5-fold cross-validation (training set: validation set = 4:1). Then, Experiment 1 is designed to evaluate the preoperative prediction models based on nine classic machine learning predictors and 125 × *N* selected radiomics features generated by the LASSO/125 × *M* (*M* = 10,20,30,40,50,60), fused radiomics features generated by PCA/ICA/UMAP/SPECTRAL with the 5-fold cross-validation. Finally, Experiment 3 is designed to evaluate the preoperative prediction models based on the nine classic machine learning predictors and 125 × (*N* + *M*) selected and fused radiomics features with the 5-fold cross-validation. A standard *z*-score normalization is applied to these radiomics features before training the nine machine learning predictors. To avoid data leakage, the LASSO algorithm is applied to the training set separately to select the radiomics features. Then, on the validation set, radiomics features consistent with the selected radiomics features from the training set are selected by indexing. Table 2 reports the definition of the feature selection and fused algorithm in Python 3.8. Meanwhile, Table 3 reports the definition of the nine machine learning predictors in Python 3.8.

**Table 2:** Definition of the feature selection and fused algorithm in Python 3.8.

Feature selection/Fused algorithm	Definition in Python 3.8
LASSO	alphas = np.logspace(-12, 1, 2000, base=2) lasso = LassoCV(alphas=alphas, cv=10, random_state=0, max_iter=100000).fit(X, Y)
ICA	ica = FastICA(n_components=n_components, random_state=42,max_iter=1000)
PCA	pca = PCA(n_components=n_components, random_state=42)
ISOMAP	isomap = isomap(n_components=n_components, n_neighbors=n_neighbors)
UMAP	n_neighbors_umap = min(15, features.shape[0] - 1) min_dist = 0.1 umap_reducer=umap.UMAP(n_components=n_components,n_neighbors=n_neighbors_umap,min_dist=min_dist,random_state=42,metric=‘euclidean’)
SPECTRAL	Spectral=SpectralEmbedding(n_components=n_components,n_neighbors=n_neighbors_spectral,random_state=42)

**Table 3:** Definition of the nine machine learning predictors in Python 3.8.

Predictor	Definition in Python 3.8
RF	‘RF’: RandomForestClassifier(n_estimators=100, random_state=90)
MLP	‘MLP’: MLPClassifier(hidden_layer_sizes=(400, 100), alpha=0.001, max_iter=1000, random_state=42)
KNN	‘KNN’: KNeighborsClassifier(n_neighbors=10, leaf_size=60)
SVM	‘SVM’: svm.SVC(C=1.0, kernel=‘rbf’, degree=3, gamma=‘auto’,coef0=0.0, shrinking=True, probability=True, tol=0.001, cache_size=200, class_weight=None, verbose=False, max_iter=-1, random_state=42)
LR	‘LR’: LogisticRegression(C=1.1, class_weight=None, dual=False, fit_intercept=True, intercept_scaling=1, max_iter=100000, multi_class=‘ovr’, penalty=‘l2’, random_state=42, solver=‘liblinear’, tol=0.0001, warm_start=True)
DT	‘DT’: DecisionTreeClassifier(random_state=42)
GBDT	‘GBDT’: GradientBoostingClassifier(random_state=42)
LDA	‘LDA’: LinearDiscriminantAnalysis(solver=‘eigen’, shrinkage=0.1)
Ada	‘Ada’: AdaBoostClassifier(algorithm="SAMME", n_estimators=80, learning_rate=0.75, random_state=42)

#### 3.1.1 Evaluation metrics

To assess performance differences between the preoperative prediction models, five standard evaluation metrics used in this study include the accuracy, precision, recall, *F*_1_-score, and area under the curve (AUC) (24–36). Specifically, the AUC can be calculated by the receiver operating characteristic (ROC) curve. In addition, the accuracy, precision, recall, and *F*_1_-scores are defined by Equations 1–4:


$$\text{Accuracy}=\frac{\mathrm{TP}+\mathrm{TN}}{\mathrm{TP}+\mathrm{TN}+\mathrm{FP}+\mathrm{FN}\;},$$ (1)

$$\text{Precision}=\frac{\mathrm{TP}}{\mathrm{TP}+\mathrm{FP}},$$ (2)

$$\text{Recall}=\frac{\mathrm{TP}}{\mathrm{TP}+\mathrm{FN}},$$ (3)

$$F_{1}-\text{score}=\frac{2\times \text{Precision}\times \text{Recall}}{\text{Precision}+\text{Recall}},$$ (4)

where TP and TN represent the number of positive MVIs predicted to be positive and the number of negative MVIs predicted to be negative. In contrast, FP and FN represent the number of positive MVIs predicted to be negative and the number of negative MVIs predicted to be positive.

Based on Equations 1–4 and the AUC, Equation 5 is determined.


$$\text{Mean evaluation metric}=(\begin{array}{l} \text{mean accuracy}\\ +\text{mean precision}+\text{mean recall}\\ +\text{mean}\;F_{1}-\text{score}+\text{mean}\;\mathrm{AUC} \end{array})/5,$$ (5)

where mean accuracy, mean precision, mean recall, mean *F*_1_-score, and mean AUC separately represent the average of the accuracy, precision, recall, *F*_1_-score, and AUC.

### 3.2 Results

This section reports the results of the comparative experiment to highlight the performance of the proposed MVI preoperative prediction model (RF + LASSO + SPECTRAL-10). Overall, the proposed MVI preoperative prediction model (RF + LASSO + SPECTRAL-10) achieves a mean accuracy of 0.7520 ± 0.0867, a mean precision of 0.7354 ± 0.1863, a mean recall of 0.6955 ± 0.2203, a mean *F*_1_-score of 0.6943 ± 0.1437, and a mean AUC of 0.7962 ± 0.1700.

#### 3.2.1 Comparative results of the MVI preoperative prediction models in Experiment 1

**Figure 4:**
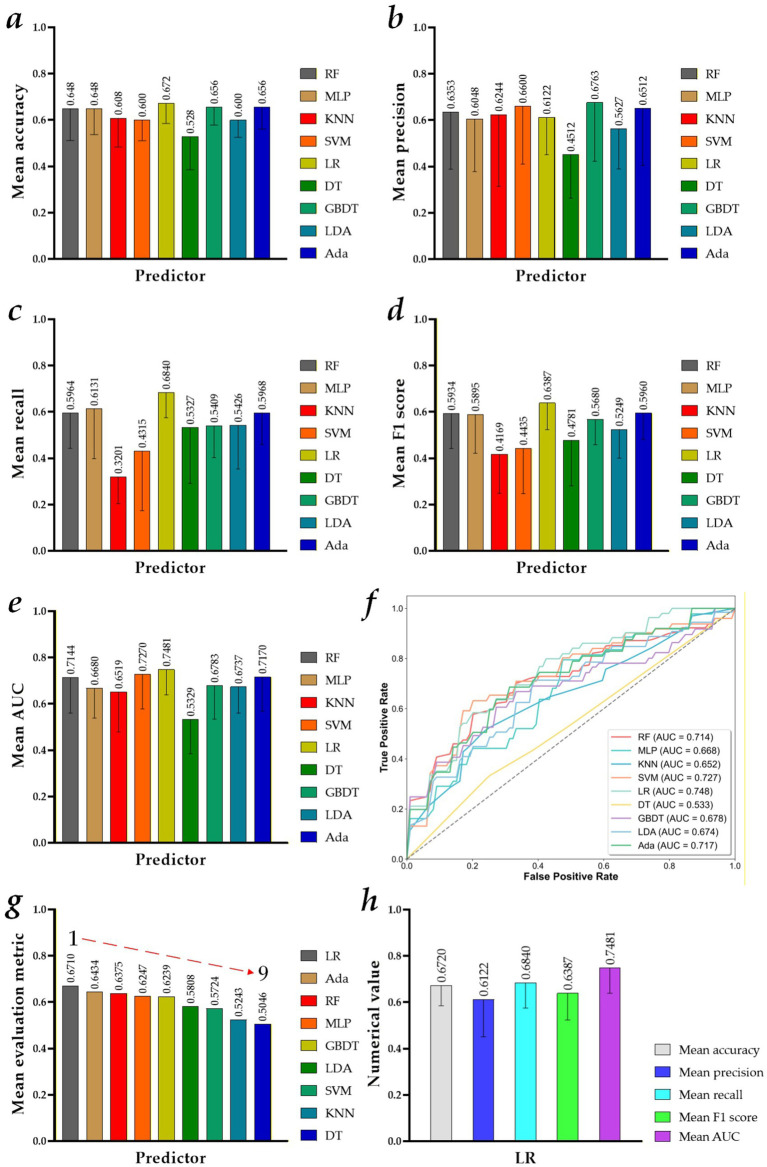
Visualized results of the microvascular invasion (MVI) preoperative prediction models constructed by different machine learning predictors and 125 × 1,692 MR Radiomics features: **(a)** mean accuracy; **(b)** mean precision; **(c)** mean recall; **(d)** mean *F*_1_-score; **(e)** mean AUC; **(f)** ROC curves of the MVI preoperative prediction models constructed by different machine learning predictors and 125 × 1,692 MR Radiomics features; **(g)** model performance ranking based on the mean evaluation metrics (machine learning predictor + 125 × 1,692 MR Radiomics features); and **(h)** mean evaluation metrics of the best performance model (LR + 125 × 1,692 MR Radiomics features).

Figure 4 shows the visualized results of the MVI preoperative prediction models constructed by different machine learning predictors and 1,692 MR Radiomics features. Specifically, the performance of these preoperative prediction models in Experiment 1 is reported in Table A1. Figures 4a–e show a mean accuracy, a mean precision, a mean recall, a mean *F*_1_-score, and a mean AUC of the MVI preoperative prediction models constructed by different machine learning predictors and 1,692 MR Radiomics features. Figure 4f shows the ROC curves of the MVI preoperative prediction models constructed by different machine learning predictors and 125 × 1,692 MR Radiomics features.

Overall, Figures 4g,h show that the MVI preoperative prediction model based on the LR and 125 × 1,692 MR Radiomics features performs the best, achieving a mean accuracy of 0.6720 ± 0.0867, a mean precision of 0.6122 ± 0.1615, a mean recall of 0.6840 ± 0.1088, a mean *F*_1_-score of 0.6387 ± 0.1151, and a mean AUC of 0.7481 ± 0.1087. In addition, compared with the mean evaluation metric of other MVI preoperative prediction models constructed by RF, MLP, KNN, SVM, DT, GBDT, LDA, and Ada and 125 × 1,692 MR Radiomics features, the best MVI preoperative prediction model improves by 3.35, 4.63, 14.67, 9.86, 16.64, 4.71, 9.02, and 2.76%, respectively.

#### 3.2.2 Comparative results of the MVI preoperative prediction models in Experiment 2

**Figure 5:**
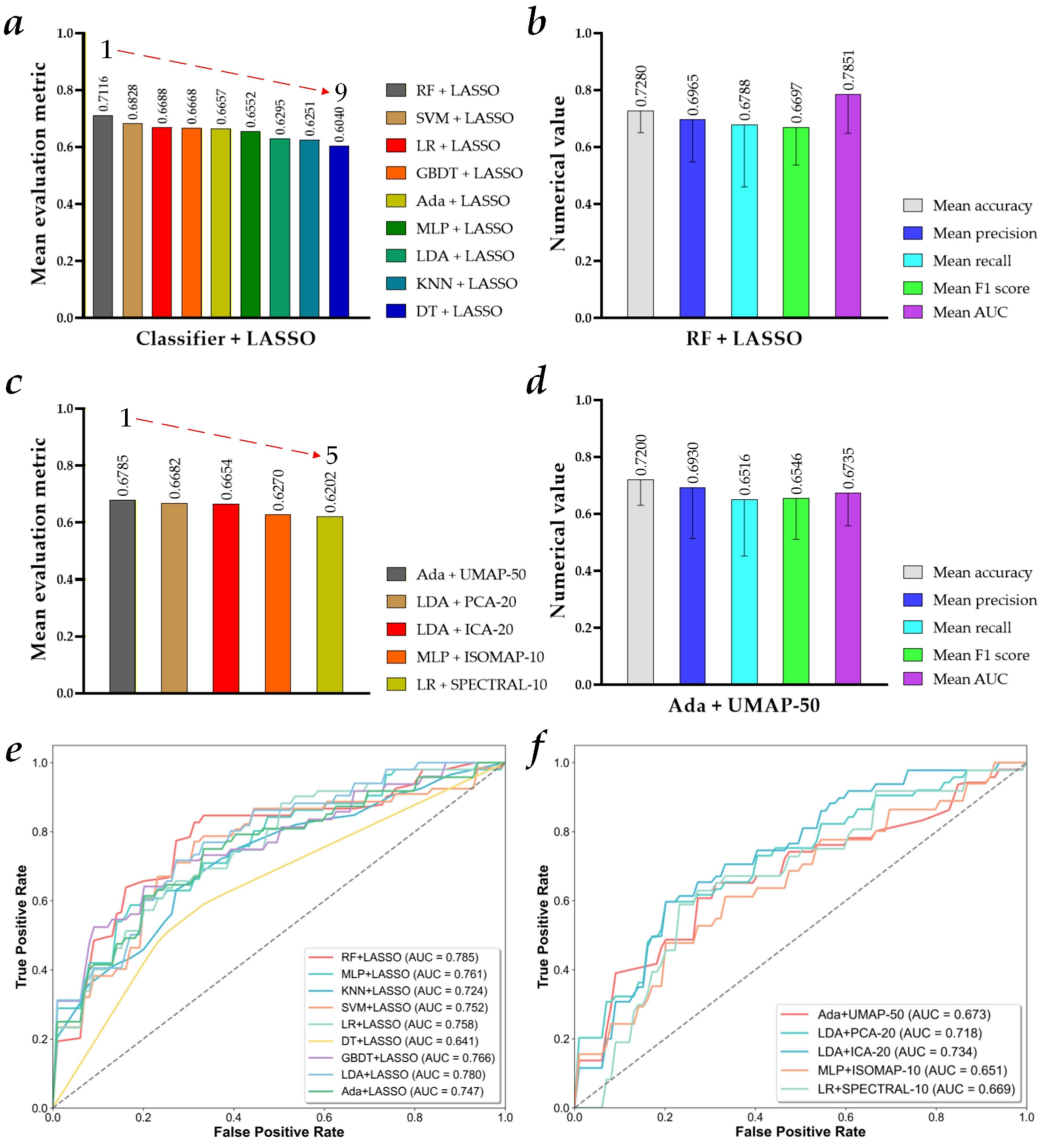
Visualized results of the microvascular invasion (MVI) preoperative prediction models constructed by different machine learning predictors with 125 × *N* selected MR Radiomics features, or 125 × *M* fused MR Radiomics features. **(a)** Model performance ranking based on the mean evaluation metrics (machine learning predictor + 125 × *N* selected MR Radiomics features); **(b)** mean evaluation metrics of the best performance model (RF + LASSO); **(c)** top five model performance ranking based on the mean evaluation metrics (machine learning predictor + 125 × *M* fused MR Radiomics features); **(d)** mean evaluation metrics of the best performance model (Ada + UMAP-50); **(e)** ROC curves of the MVI preoperative prediction models constructed by different machine learning predictors and 125 × *N* selected MR Radiomics features; and **(f)** ROC curves of the top five MVI preoperative prediction models constructed by different machine learning predictors and 125 × *M* fused MR Radiomics features.

Figure 5 shows the visualized results of the MVI preoperative prediction models constructed by different machine learning predictors with 125 × *N* selected MR Radiomics features or 125 × *M* fused MR Radiomics features. Specifically, the performance of these preoperative prediction models in Experiment 2 is reported in Table A2. Figures 5a,b,e show the model performance ranking based on the mean evaluation metrics (machine learning predictor + 125 × *N* selected MR Radiomics features), the mean evaluation metrics of the best performance model (RF + LASSO), and their ROC curves. Meanwhile, Figures 5c,d,f show the model performance ranking based on the mean evaluation metrics (machine learning predictor + 125 × *M* fused MR Radiomics features), the mean evaluation metrics of the best performance model (Ada + UMAP-50), and their ROC curves.

First, the MVI preoperative prediction model based on the RF and 125 × *N* selected MR Radiomics features using the LASSO algorithm (RF + LASSO) performs the best among all machine learning predictors using the LASSO algorithm, achieving a mean accuracy of 0.7280 ± 0.0769, a mean precision of 0.6965 ± 0.1481, a mean recall of 0.6788 ± 0.2183, a mean *F*_1_-score of 0.6697 ± 0.1324, and a mean AUC of 0.7851 ± 0.1363. In addition, compared with the mean evaluation metric of other MVI preoperative prediction models constructed by MLP, KNN, SVM, LR, DT, GBDT, LDA, and Ada and 125 × *N* selected radiomics features, the best MVI preoperative prediction model improves by 5.64, 8.65, 2.88, 4.28, 10.76, 4.48, 8.21, and 4.59%, respectively. Compared to the mean evaluation metric of Experiment 1, the mean evaluation metric of the MVI preoperative prediction models constructed by RF, MLP, KNN, SVM, LR, DT, GBDT, LDA, and Ada and 125 × *N* selected radiomics features in Experiment 2 improves by 7.41, 3.05, 10.09, 11.04, −0.22%, 9.94, 4.29, 4.87, and 2.23%, respectively.

Second, the MVI preoperative prediction model based on the Ada and 125 × 50 fused MR Radiomics features using the UMAP algorithm (Ada + UMAP-50) performs the best among all machine learning predictors using the ICA, PCA, ISOMAP, UMAP, and SPECTRAL algorithms, achieving a mean accuracy of 0.7200 ± 0.0894, a mean precision of 0.6930 ± 0.1795, a mean recall of 0.6516 ± 0.1992, a mean *F*_1_-score of 0.6546 ± 0.1434, and a mean AUC of 0.6735 ± 0.1145. In addition, compared to the mean evaluation metric of other best MVI preoperative prediction models among all machine learning predictors using each feature fusion algorithm, LDA + PCA-20, LDA + ICA-20, MLP + ISOMAP-10, and LR + SPECTRAL-10, mean evaluation metric of Ada + UMAP-50 model improves by 1.03, 1.31, 5.51, and 5.38%, respectively. Compared to the mean evaluation metric of the MVI preoperative prediction model based on Ada and 125 × 1,692 MR Radiomics features in Experiment 1, the mean evaluation metric of Ada + UMAP-50 model in Experiment 2 improves by 3.15%. Compared with the mean evaluation metric of the MVI preoperative prediction model based on LDA and 125 × 1,692 MR Radiomics features in Experiment 1, the mean evaluation metric of the LDA + PCA-20 and LDA + ICA-20 models in Experiment 2 improves by 8.74 and 8.46%, respectively. Compared to the mean evaluation metric of the MVI preoperative prediction model based on MLP and 125 × 1,692 MR Radiomics features in Experiment 1, the mean evaluation metric of MLP + ISOMAP-10 model in Experiment 2 improves by 0.23%.

#### 3.2.3 Comparative results of the MVI preoperative prediction models in Experiment 3

**Figure 6:**
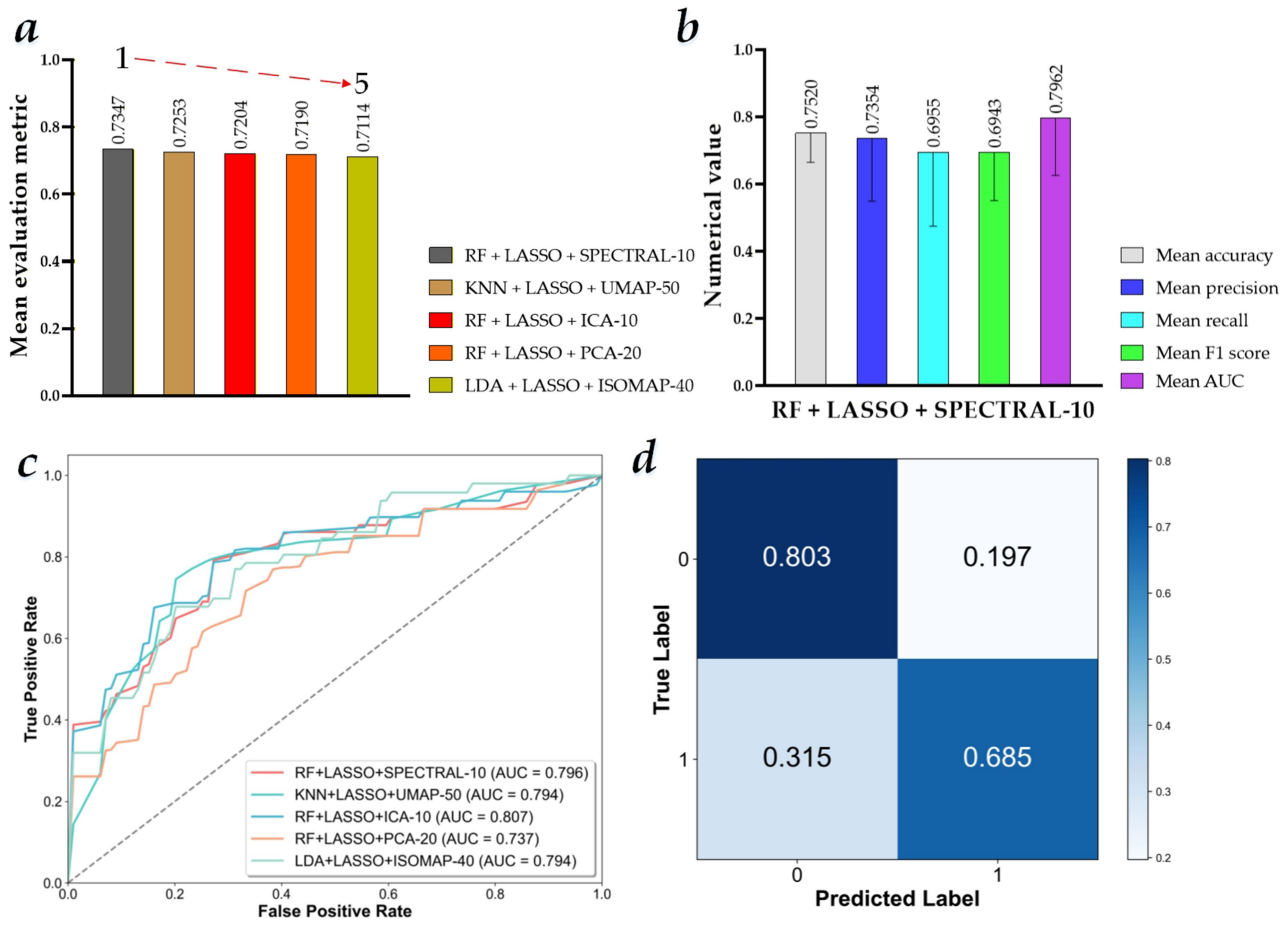
Visualized results of the microvascular invasion (MVI) preoperative prediction models constructed by different machine learning predictors with 125 × *N* selected MR Radiomics features and 125 × *M* fused MR Radiomics features. **(a)** Top five model performance ranking based on the mean evaluation metrics (machine learning predictor + 125 × *N* selected MR Radiomics features + 125 × *M* fused MR Radiomics features); **(b)** mean evaluation metrics of the best performance model (RF + LASSO + SPECTRAL-10); **(c)** ROC curves of the top five MVI preoperative prediction models constructed by different machine learning predictors with 125 × *N* selected MR Radiomics features and 125 × *M* fused MR Radiomics features; and **(d)** confusion matrix of the best performance model (RF + LASSO + SPECTRAL-10).

Figure 6 shows the visualized results of the MVI preoperative prediction models constructed by different machine learning predictors with 125 × *N* selected MR Radiomics features and 125 × *M* fused MR Radiomics features. Specifically, the performance of these preoperative prediction models in Experiment 3 is reported in Table A3. Figures 6a–d show the model performance ranking based on the mean evaluation metrics (machine learning predictor + 125 × *N* selected MR Radiomics features + 125 × *M* fused MR Radiomics features), the mean evaluation metrics of the best performance model (KNN + LASSO + UMAP-50), their ROC curves, and confusion matrix of the best performance model.

The MVI preoperative prediction model based on the RF with 125 × *N* selected MR Radiomics features and 125 × 10 fused MR Radiomics features (RF + LASSO + ICA-10) performs the best among all machine learning predictors using LASSO and ICA, PCA, ISOMAP, UMAP, and SPECTRAL algorithms, achieving a mean accuracy of 0.7520 ± 0.0867, a mean precision of 0.7354 ± 0.1863, a mean recall of 0.6955 ± 0.2203, a mean *F*_1_-score of 0.6943 ± 0.1437, and a mean AUC of 0.7962 ± 0.1700. In addition, compared to the mean evaluation metric of other best MVI preoperative prediction models among all machine learning predictors using the LASSO and each feature fusion algorithm, KNN + LASSO + UMAP-50, RF + LASSO + ICA-10, RF + LASSO + PCA-20, and LDA + LASSO + ISOMAP-40, mean evaluation metric of RF + LASSO + ICA-10 model improves by 0.94, 1.43, 1.57, and 2.33%, respectively. Compared to the mean evaluation metric of RF + LASSO, the mean evaluation metric of RF + LASSO + ICA-10, RF + LASSO + ICA-10, and RF + LASSO + PCA-20 models improves by 2.31, 0.87, and 0.73%, respectively. Compared with the mean evaluation metric for KNN + LASSO, the mean evaluation metric of KNN + LASSO + UMAP-50 model improves by 10.02%. Compared to the mean evaluation metric of LDA + LASSO, the mean evaluation metric of LDA + LASSO + ISOMAP-40 model improves by 8.19%.

#### 3.2.4 DeLong’s test

To compare whether there are significant differences in the areas under the two ROC curves of the top five models in Experiment 3, DeLong’s test (45, 46), a non-parametric statistical method, is performed using Statistical Product and Service Solutions (SPSS, launched by IBM, version 27).

Table 4 reports the paired-sample area difference under the ROC curves based on DeLong’s test. Specifically, compared to the significance among the MVI preoperative prediction models constructed by RF vs. RF + LASSO, the significance among the MVI preoperative prediction models constructed by RF + LASSO vs. RF + LASSO + PECTRAL-10/RF vs. RF + LASSO + ICA-10 improves by 0.30%/0.50%. Compared to the significance among the MVI preoperative prediction models constructed by KNN vs. KNN + LASSO, the significance among the MVI preoperative prediction models constructed by KNN vs. KNN + LASSO + UMAP-50 improves by 5.7%. Compared to the significance among the MVI preoperative prediction models constructed by LDA vs. LDA + LASSO, the significance among the MVI preoperative prediction models constructed by LDA vs. LDA + LASSO + ISOMAP-40 improves by 2.2%.

**Table 4:** Paired-sample area difference under the ROC curves based on DeLong’s test.

Test result pair(s)	Asymptotic		AUC difference	Std. error difference^b^	Asymptotic 95% confidence interval	
	*z*	Sig. (2-tail)^a^			Lower bound	Upper bound
RF vs. RF + LASSO	−2.809	0.005	−0.071	0.301	−1.21	−0.021
RF vs. RF + LASSO + SPECTRAL-10	−3.075	0.002	−0.072	0.302	−0.118	−0.026
RF vs. RF + LASSO + UMAP-50	−1.906	0.057	−0.038	0.304	−0.077	0.001
RF vs. RF + LASSO + ICA-10	−3.544	0.000 (<0.001)	−0.092	0.299	−0.143	−0.041
RF vs. RF + LASSO + PCA-20	−2.437	0.015	−0.060	0.302	−0.108	−0.012
KNN vs. KNN + LASSO	−1.901	0.057	−0.079	0.308	−0.160	0.002
KNN vs. KNN + LASSO + UMAP-50	−4.290	0.000 (<0.001)	−0.137	0.303	−0.200	−0.074
LDA vs. LDA + LASSO	−2.157	0.031	−0.099	0.301	−0.189	−0.009
LDA vs. LDA + LASSO + ISOMAP-40	−2.621	0.009	−0.127	0.297	−0.222	−0.032

a Null hypothesis: true area difference = 0.

b Under the non-parametric assumption.

## 4 Discussion

This section conducts the following discussions based on the experimental results. In addition, this section outlines the limitations of this study and its future direction.

### 4.1 RF predictor

The core advantage of random forest lies in its high robustness and generalization ability, enabled by dual randomness, making it the mainstream algorithm for processing small- to medium-sized data (47), such as the 125 cases of MR Radiomics features. First, by using bootstrap sampling and random selection of MR Radiomics features, inter-tree correlation can be reduced, thereby helping the MVI preoperative prediction model avoid excessive dependence on noise. Second, independent generation across trees supports distributed computing and yields significantly faster training than serial algorithms. Finally, by using self-sampling and majority voting, the problem of category bias can be alleviated, which is particularly important for constructing the MVI preoperative prediction model.

### 4.2 SPECTRAL

The advantage of SPECTRAL lies in capturing the intrinsic topological structure of data via the spectral decomposition of the Turalaplus matrix, which is particularly suitable for low-dimensional embedding of non-linear, high-dimensional data (40, 41), such as the 25 × 1,692 MR Radiomics features of HCC in each cross-validation fold. First, a similarity matrix (e.g., a Gaussian kernel) is constructed using graph theory, and the 25 × 1,692 MR Radiomics features of HCC are mapped to a low-dimensional space via eigenvalue decomposition, overcoming the limitations of linear dimensionality reduction. In addition, it takes into account both the local neighborhood relationships, as captured by the similarity matrix, and the global topology of the Laplacian matrix, as encoded in the feature vector, to capture the overall connectivity.

### 4.3 Optimized MR Radiomics combination strategy

The proposed optimized MR Radiomics combination strategy is similar to neural architecture search (48, 49), which determines the optimal network architecture for classification and segmentation tasks. The proposed optimized MR Radiomics combination strategy combines linearly selected MR Radiomics features and non-linearly fused MR Radiomics features and concatenates them to generate an optimized MR Radiomics combination vector that matches the predictor. Since the optimized MR Radiomics combination vector includes both linearly selected MR Radiomics features and non-linearly fused MR Radiomics features, it facilitates RF predictor sampling and feature random selection, thereby reducing inter-tree correlation and avoiding excessive dependence on noise in the MVI preoperative prediction model. In addition, non-linear MR Radiomics features are fused from all MR Radiomics of each case to avoid the problem that LASSO’s selection may miss important non-linear MR Radiomics features.

### 4.4 Proposed MVI preoperative prediction model

The proposed MVI preoperative prediction model (RF + LASSO + SPECTRAL-10) only needs to extract MR features from abdominal enhanced T1WI images during the artistic phase, without the need for T2-weighted, diffusion-weighted, or other types of images, and without the need to collect non-imaging information of enhanced MRI (such as aminotransferase-to-platelet ratio and gamma-glutamyl transferase-to-platelet ratio) (38). The proposed MVI preoperative prediction model (RF + LASSO + SPECTRAL-10) also avoids the highly subjective determination of the perioperative region. Therefore, the proposed MVI preoperative prediction model (RF + LASSO + SPECTRAL-10) simplifies its clinical application. In addition, compared with the previous contrast agent, Gd-EOB-DTPA (39), the price of the Gd-DTPA contrast agents used in this study is lower, thereby reducing the financial burden of the disease. Meanwhile, the arterial phase images of Gd-DTPA contrast agents are relatively less susceptible to respiratory motion artifacts and maintain stable quality.

### 4.5 Limitations and future studies

Although we propose an MVI preoperative prediction model for MVI in HCC to eliminate non-imaging information of enhanced MRI and the highly subjective determination of perioperative region, HCC mask images are manually segmented by radiologists. This may hinder the clinical application of the MVI preoperative prediction model. Therefore, the subsequent research aims to develop an HCC automatic segmentation model to fully automate the prediction process. In addition, we do not have sufficient abdominal enhanced MRI images to further validate the proposed MVI preoperative prediction model’s performance. Therefore, we encourage researchers to collect more abdominal-enhanced MRI images to validate the performance of the proposed MVI preoperative prediction models. Finally, the engineering of the constructed prediction model should also be considered for clinical practice.

## 5 Conclusion

To address the problem of combining non-imaging information from enhanced MRI with the highly subjective determination of the perioperative region, we propose an enhanced preoperative prediction model for MVI in HCC using an optimized MR Radiomics combination strategy and a machine learning predictor. First, the HCC was manually segmented from 125 × 512 × 512 × *N* abdominal enhanced T1WI images during the arterial phase, generating 125 × 512 × 512 × *N* HCC mask images. Second, 125 × 1,692 MR Radiomics features of HCC are extracted from abdominal enhanced T1WI images based on the HCC mask images. Third, the 125 × *N* selected and 125 × 10 fused MR Radiomics features are determined using the proposed optimized MR Radiomics combination strategy with 5-fold cross-validation. Finally, the best preoperative prediction model is constructed using a random forest (RF) predictor with 125 × *N* selected and 125 × 10 fused MR Radiomics features. The results show that the proposed MVI preoperative prediction model (RF + LASSO + SPECTRAL-10) achieves a mean accuracy of 0.7520 ± 0.0867, a mean precision of 0.7354 ± 0.1863, a mean recall of 0.6955 ± 0.2203, a mean *F*_1_-score of 0.6943 ± 0.1437, and a mean AUC of 0.7962 ± 0.1700. The proposed best preoperative prediction model can effectively predict MVI in HCC, potentially serving as a strong decision-making tool for these vulnerable populations.

## Data Availability

The raw data supporting the conclusions of this article will be made available by the authors, without undue reservation.
