# Supplementary material for: Enhanced preoperative prediction for microvascular invasion in hepatocellular carcinoma through an optimized MR Radiomics combination strategy and machine learning predictor
Source: Front Med (Lausanne). 2026 Feb 11;13:1764733. doi: 10.3389/fmed.2026.1764733 (PMC12932187; doi:10.3389/fmed.2026.1764733)
Supplement: Supplementary file 2 [file Table_1.docx]

Appendix

**Table A1.** The performance of the preoperative prediction model in Experiment 1.

| **Predictor** | **Evaluation metrics** | | | | |
| --- | --- | --- | --- | --- | --- |
|  | Mean accuracy ± SD | Mean precision ± SD | Mean recall ± SD | Mean F1-score ± SD | Mean AUC ± SD |
| RF | 0.6480±0.1368 | 0.6353±0.2468 | 0.5964±0.1538 | 0.5934±0.1510 | 0.7144±0.1545 |
| MLP | 0.6480±0.1110 | 0.6048±0.2273 | 0.6131±0.2145 | 0.5895±0.1676 | 0.6680±0.1293 |
| KNN | 0.6080±0.1246 | 0.6244±0.3101 | 0.3201±0.1159 | 0.4169±0.1682 | 0.6519±0.1732 |
| SVM | 0.6000±0.0894 | 0.6600±0.2496 | 0.4315±0.2574 | 0.4435±0.1962 | 0.7270±0.1490 |
| LR | 0.6720±0.0867 | 0.6122±0.1615 | 0.6840±0.1088 | 0.6387±0.1151 | 0.7481±0.1087 |
| DT | 0.5280±0.1425 | 0.4512±0.1883 | 0.5327±0.2412 | 0.4781±0.1975 | 0.5329±0.1477 |
| GBDT | 0.6560±0.0780 | 0.6763±0.2535 | 0.5409±0.1377 | 0.5680±0.1092 | 0.6783±0.1446 |
| LDA | 0.6000±0.0748 | 0.5627±0.1736 | 0.5426±0.1894 | 0.5249±0.1245 | 0.6737±0.1135 |
| Ada | 0.6560±0.0963 | 0.6512±0.2470 | 0.5968±0.1374 | 0.5960±0.1148 | 0.7170±0.1485 |

**Table A2.** The performance of the preoperative prediction model in Experiment 2.

| **Predictor + Features** | **Evaluation metrics** | | | | |
| --- | --- | --- | --- | --- | --- |
|  | Mean accuracy ± SD | Mean precision ± SD | Mean recall ± SD | Mean F1-score ± SD | Mean AUC ± SD |
| RF + LASSO | 0.7280±0.0769 | 0.6965±0.1481 | 0.6788±0.2183 | 0.6697±0.1324 | 0.7851±0.1363 |
| MLP + LASSO | 0.6560±0.0358 | 0.5945±0.0974 | 0.6497±0.1081 | 0.6147±0.0697 | 0.7610±0.1009 |
| KNN + LASSO | 0.6800±0.0894 | 0.6728±0.1992 | 0.4877±0.1287 | 0.5609±0.1499 | 0.7242±0.1229 |
| SVM + LASSO | 0.7120±0.0335 | 0.6774±0.1007 | 0.6264±0.0918 | 0.6466±0.0771 | 0.7515±0.1200 |
| LR + LASSO | 0.6960±0.0537 | 0.6753±0.1511 | 0.5931±0.1170 | 0.6210±0.0952 | 0.7584±0.0896 |
| DT + LASSO | 0.6560±0.0727 | 0.5921±0.1489 | 0.5607±0.1799 | 0.5696±0.1604 | 0.6414±0.0918 |
| GBDT + LASSO | 0.6960±0.1081 | 0.6911±0.2532 | 0.5699±0.1642 | 0.6109±0.1643 | 0.7662±0.1768 |
| LDA + LASSO | 0.6640±0.0607 | 0.6257±0.1361 | 0.5155±0.1228 | 0.5626±0.1221 | 0.7797±0.0944 |
| Ada + LASSO | 0.6880±0.0955 | 0.6741±0.2227 | 0.6091±0.2348 | 0.6101±0.1578 | 0.7472±0.1759 |
| RF + ICA-10 | 0.6800±0.0849 | 0.6495±0.1885 | 0.5888±0.0970 | 0.6099±0.1197 | 0.7152±0.1162 |
| MLP + ICA-10 | 0.6240±0.0780 | 0.5946±0.1372 | 0.5277±0.1257 | 0.5425±0.0866 | 0.6566±0.0961 |
| KNN + ICA-10 | 0.5840±0.0921 | 0.5454±0.2352 | 0.3826±0.1344 | 0.4373±0.1508 | 0.6169±0.1309 |
| SVM + ICA-10 | 0.6640±0.1004 | 0.6667±0.2379 | 0.5414±0.1068 | 0.5795±0.1266 | 0.7228±0.1279 |
| LR + ICA-10 | 0.6000±0.1356 | 0.5577±0.2278 | 0.4771±0.2171 | 0.4942±0.1895 | 0.6304±0.1622 |
| DT + ICA-10 | 0.5840±0.0607 | 0.5389±0.1097 | 0.5456±0.0849 | 0.5289±0.0219 | 0.5860±0.0449 |
| GBDT + ICA-10 | 0.6800±0.0800 | 0.6489±0.1502 | 0.5531±0.1048 | 0.5953±0.1177 | 0.7148±0.1241 |
| LDA + ICA-10 | 0.6000±0.1356 | 0.5577±0.2278 | 0.4771±0.2171 | 0.4942±0.1895 | 0.6236±0.1658 |
| Ada + ICA-10 | 0.6640±0.0537 | 0.6175±0.0929 | 0.5953±0.1140 | 0.6003±0.0707 | 0.6945±0.0774 |
| RF + ICA-20 | 0.5600±0.0938 | 0.4843±0.1382 | 0.3226±0.1940 | 0.3659±0.1822 | 0.5913±0.1062 |
| MLP + ICA-20 | 0.5920±0.1213 | 0.5388±0.2090 | 0.4997±0.1484 | 0.5100±0.1543 | 0.6007±0.1603 |
| KNN + ICA-20 | 0.5920±0.0438 | 0.5151±0.2496 | 0.2825±0.1620 | 0.3563±0.1897 | 0.6139±0.0826 |
| SVM + ICA-20 | 0.6800±0.0566 | 0.7273±0.2551 | 0.4823±0.1366 | 0.5573±0.1078 | 0.7125±0.1154 |
| LR + ICA-20 | 0.6560±0.0669 | 0.6212±0.1749 | 0.5931±0.0607 | 0.5962±0.0849 | 0.7135±0.0741 |
| DT + ICA-20 | 0.5440±0.0727 | 0.4888±0.1070 | 0.5423±0.0825 | 0.5045±0.0598 | 0.5500±0.0551 |
| GBDT + ICA-20 | 0.5760±0.0456 | 0.5204±0.0891 | 0.3982±0.1462 | 0.4355±0.0938 | 0.5793±0.0332 |
| LDA + ICA-20 | 0.6880±0.0716 | 0.6476±0.1659 | 0.6262±0.0658 | 0.6311±0.1014 | 0.7342±0.0717 |
| Ada + ICA-20 | 0.5760±0.0219 | 0.5191±0.0776 | 0.4547±0.1161 | 0.4728±0.0534 | 0.6357±0.0448 |
| RF + ICA-30 | 0.6480±0.0867 | 0.6100±0.2094 | 0.5020±0.2164 | 0.5336±0.1775 | 0.6442±0.1793 |
| MLP + ICA-30 | 0.6000±0.0566 | 0.5383±0.1450 | 0.5388±0.1782 | 0.5259±0.1240 | 0.6506±0.0739 |
| KNN + ICA-30 | 0.6080±0.0438 | 0.5967±0.1894 | 0.2373±0.0898 | 0.3370±0.1188 | 0.5808±0.1909 |
| SVM + ICA-30 | 0.6080±0.0335 | 0.6709±0.2269 | 0.3438±0.1315 | 0.4154±0.0785 | 0.6567±0.1083 |
| LR + ICA-30 | 0.6240±0.0456 | 0.5582±0.1025 | 0.6032±0.2373 | 0.5616±0.1278 | 0.6784±0.0799 |
| DT + ICA-30 | 0.6160±0.0669 | 0.5662±0.1099 | 0.5244±0.1154 | 0.5366±0.0853 | 0.6091±0.0687 |
| GBDT + ICA-30 | 0.5840±0.0727 | 0.5183±0.1875 | 0.4353±0.1520 | 0.4637±0.1522 | 0.5914±0.1238 |
| LDA + ICA-30 | 0.6560±0.0456 | 0.6156±0.1110 | 0.5610±0.1875 | 0.5709±0.1130 | 0.6955±0.0914 |
| Ada + ICA-30 | 0.6800±0.0748 | 0.6215±0.1501 | 0.6627±0.1541 | 0.6317±0.1267 | 0.6816±0.1285 |
| RF + ICA-40 | 0.6240±0.0607 | 0.7184±0.2663 | 0.4708±0.2297 | 0.4905±0.1317 | 0.5984±0.0643 |
| MLP + ICA-40 | 0.6400±0.0632 | 0.6014±0.1728 | 0.6321±0.1061 | 0.5996±0.0735 | 0.7209±0.0550 |
| KNN + ICA-40 | 0.5600±0.0632 | 0.5167±0.2911 | 0.1919±0.1284 | 0.2590±0.1286 | 0.5697±0.1219 |
| SVM + ICA-40 | 0.5760±0.0780 | 0.6408±0.3290 | 0.3617±0.1092 | 0.4175±0.0647 | 0.6559±0.0825 |
| LR + ICA-40 | 0.6480±0.0593 | 0.5848±0.0946 | 0.6821±0.1961 | 0.6160±0.0909 | 0.7371±0.0814 |
| DT + ICA-40 | 0.4720±0.0593 | 0.4010±0.0943 | 0.3993±0.1195 | 0.3881±0.0692 | 0.4720±0.0596 |
| GBDT + ICA-40 | 0.5520±0.0522 | 0.4881±0.0745 | 0.4971±0.2025 | 0.4727±0.0970 | 0.5735±0.0939 |
| LDA + ICA-40 | 0.6400±0.0490 | 0.5855±0.1049 | 0.6412±0.1555 | 0.5980±0.0771 | 0.7174±0.0436 |
| Ada + ICA-40 | 0.5840±0.0537 | 0.5196±0.0443 | 0.6349±0.2568 | 0.5488±0.1082 | 0.6229±0.1164 |
| RF + ICA-50 | 0.4560±0.0607 | 0.3319±0.1459 | 0.2198±0.1941 | 0.2313±0.1360 | 0.4464±0.1340 |
| MLP + ICA-50 | 0.6560±0.1004 | 0.6557±0.2547 | 0.6186±0.1226 | 0.6073±0.1035 | 0.6862±0.1417 |
| KNN + ICA-50 | 0.5920±0.0867 | 0.6750±0.3092 | 0.2987±0.2218 | 0.3549±0.1815 | 0.6542±0.1832 |
| SVM + ICA-50 | 0.6160±0.0607 | 0.7300±0.2775 | 0.3473±0.1336 | 0.4260±0.0592 | 0.5475±0.1999 |
| LR + ICA-50 | 0.6320±0.0593 | 0.6107±0.1647 | 0.6058±0.1599 | 0.5783±0.0699 | 0.7034±0.0823 |
| DT + ICA-50 | 0.5280±0.1425 | 0.4579±0.2189 | 0.3926±0.1887 | 0.4134±0.1876 | 0.5166±0.1484 |
| GBDT + ICA-50 | 0.4960±0.0607 | 0.4179±0.0934 | 0.3652±0.1492 | 0.3703±0.1056 | 0.4968±0.1293 |
| LDA + ICA-50 | 0.6400±0.0748 | 0.5871±0.0952 | 0.5858±0.1813 | 0.5733±0.1116 | 0.6872±0.1195 |
| Ada + ICA-50 | 0.4800±0.1327 | 0.3687±0.1632 | 0.4154±0.3077 | 0.3725±0.2186 | 0.5442±0.1023 |
| RF + ICA-60 | 0.5600±0.0800 | 0.5144±0.1899 | 0.3030±0.1282 | 0.3620±0.1241 | 0.4957±0.1233 |
| MLP + ICA-60 | 0.6080±0.0867 | 0.5646±0.1309 | 0.5738±0.1687 | 0.5494±0.1031 | 0.6306±0.0824 |
| KNN + ICA-60 | 0.5840±0.0607 | 0.6267±0.3419 | 0.1865±0.1031 | 0.2698±0.1439 | 0.5703±0.1156 |
| SVM + ICA-60 | 0.5760±0.0607 | 0.6655±0.3363 | 0.2252±0.0546 | 0.3115±0.0594 | 0.5017±0.1315 |
| LR + ICA-60 | 0.6080±0.0335 | 0.5624±0.1078 | 0.6091±0.1482 | 0.5645±0.0506 | 0.6660±0.0851 |
| DT + ICA-60 | 0.4560±0.1252 | 0.4002±0.2622 | 0.3434±0.0920 | 0.3584±0.1464 | 0.4462±0.1367 |
| GBDT + ICA-60 | 0.5360±0.0607 | 0.4692±0.1520 | 0.3806±0.1591 | 0.4016±0.1200 | 0.5429±0.1053 |
| LDA + ICA-60 | 0.6240±0.0669 | 0.5679±0.1146 | 0.6353±0.1071 | 0.5900±0.0692 | 0.6578±0.0964 |
| Ada + ICA-60 | 0.5200±0.0800 | 0.4242±0.2131 | 0.3668±0.1752 | 0.3846±0.1717 | 0.5103±0.0816 |
| RF + PCA-10 | 0.6400±0.1327 | 0.6305±0.2935 | 0.5486±0.1240 | 0.5695±0.1662 | 0.6807±0.1736 |
| MLP + PCA-10 | 0.6320±0.0769 | 0.6017±0.1485 | 0.5244±0.0912 | 0.5498±0.0811 | 0.6581±0.1139 |
| KNN + PCA-10 | 0.5760±0.0727 | 0.5321±0.2088 | 0.2973±0.1244 | 0.3710±0.1314 | 0.6171±0.1216 |
| SVM + PCA-10 | 0.6240±0.0358 | 0.6909±0.2828 | 0.4380±0.1222 | 0.4911±0.0563 | 0.7353±0.1618 |
| LR + PCA-10 | 0.6000±0.1356 | 0.5577±0.2278 | 0.4771±0.2171 | 0.4942±0.1895 | 0.6342±0.1691 |
| DT + PCA-10 | 0.6400±0.1095 | 0.5955±0.2054 | 0.6120±0.1375 | 0.5918±0.1383 | 0.6429±0.1136 |
| GBDT + PCA-10 | 0.6560±0.1004 | 0.6385±0.2265 | 0.5931±0.0607 | 0.5993±0.1101 | 0.6912±0.1636 |
| LDA + PCA-10 | 0.6160±0.1284 | 0.5782±0.2202 | 0.4771±0.2171 | 0.5032±0.1871 | 0.6247±0.1764 |
| Ada + PCA-10 | 0.6320±0.1308 | 0.5605±0.1419 | 0.5560±0.2335 | 0.5488±0.1828 | 0.6671±0.1569 |
| RF + PCA-20 | 0.6080±0.1145 | 0.5970±0.2667 | 0.4590±0.1010 | 0.5056±0.1395 | 0.6725±0.1174 |
| MLP + PCA-20 | 0.6000±0.1414 | 0.5750±0.2468 | 0.5175±0.1022 | 0.5309±0.1385 | 0.6262±0.1003 |
| KNN + PCA-20 | 0.5920±0.0335 | 0.5643±0.1083 | 0.3395±0.0990 | 0.4098±0.0566 | 0.6514±0.0700 |
| SVM + PCA-20 | 0.6800±0.0400 | 0.7432±0.2367 | 0.5114±0.1033 | 0.5750±0.0137 | 0.6859±0.1029 |
| LR + PCA-20 | 0.6640±0.0358 | 0.6301±0.1455 | 0.5755±0.0759 | 0.5929±0.0641 | 0.7161±0.0642 |
| DT + PCA-20 | 0.5280±0.0867 | 0.4545±0.1303 | 0.4369±0.1491 | 0.4369±0.1263 | 0.5150±0.0848 |
| GBDT + PCA-20 | 0.6160±0.0669 | 0.5847±0.1810 | 0.5234±0.1138 | 0.5366±0.0885 | 0.6431±0.0954 |
| LDA + PCA-20 | 0.6960±0.0669 | 0.6834±0.2034 | 0.6109±0.0545 | 0.6329±0.0839 | 0.7179±0.0823 |
| Ada + PCA-20 | 0.6080±0.0657 | 0.5746±0.1683 | 0.5056±0.0989 | 0.5239±0.0719 | 0.6354±0.0688 |
| RF + PCA-30 | 0.6720±0.0820 | 0.6646±0.2200 | 0.5377±0.0989 | 0.5835±0.1150 | 0.6788±0.1171 |
| MLP + PCA-30 | 0.6160±0.0456 | 0.5636±0.1241 | 0.5810±0.1553 | 0.5576±0.0825 | 0.6661±0.0662 |
| KNN + PCA-30 | 0.5920±0.0438 | 0.6333±0.2173 | 0.2287±0.1079 | 0.3117±0.1176 | 0.5858±0.1154 |
| SVM + PCA-30 | 0.6320±0.0179 | 0.6777±0.2038 | 0.4226±0.1954 | 0.4761±0.0951 | 0.6619±0.1272 |
| LR + PCA-30 | 0.6320±0.0522 | 0.5749±0.1168 | 0.6101±0.2702 | 0.5658±0.1341 | 0.7066±0.0602 |
| DT + PCA-30 | 0.5520±0.1397 | 0.4907±0.2132 | 0.4766±0.1815 | 0.4760±0.1765 | 0.5437±0.1482 |
| GBDT + PCA-30 | 0.6160±0.1345 | 0.5826±0.2666 | 0.4921±0.1432 | 0.5232±0.1822 | 0.6033±0.1921 |
| LDA + PCA-30 | 0.6400±0.0400 | 0.6091±0.1300 | 0.5456±0.1929 | 0.5524±0.1004 | 0.7058±0.0909 |
| Ada + PCA-30 | 0.6080±0.0912 | 0.5509±0.1705 | 0.5099±0.1023 | 0.5262±0.1312 | 0.6056±0.1089 |
| RF + PCA-40 | 0.5840±0.1081 | 0.5550±0.2185 | 0.3563±0.1633 | 0.4124±0.1731 | 0.5883±0.1217 |
| MLP + PCA-40 | 0.6080±0.0593 | 0.5603±0.1461 | 0.5568±0.1287 | 0.5441±0.0939 | 0.7003±0.0658 |
| KNN + PCA-40 | 0.5840±0.0876 | 0.5690±0.1337 | 0.1954±0.1161 | 0.2762±0.1349 | 0.5830±0.1119 |
| SVM + PCA-40 | 0.6160±0.0219 | 0.6600±0.2302 | 0.3617±0.0833 | 0.4410±0.0391 | 0.6808±0.1092 |
| LR + PCA-40 | 0.6480±0.0522 | 0.5941±0.1079 | 0.6821±0.1961 | 0.6161±0.0786 | 0.7493±0.0714 |
| DT + PCA-40 | 0.5200±0.0849 | 0.4591±0.1281 | 0.4979±0.0536 | 0.4726±0.0868 | 0.5194±0.0788 |
| GBDT + PCA-40 | 0.5840±0.1081 | 0.5633±0.2615 | 0.5142±0.0797 | 0.5186±0.1164 | 0.5816±0.1893 |
| LDA + PCA-40 | 0.6400±0.0693 | 0.5923±0.1394 | 0.5990±0.1644 | 0.5805±0.1073 | 0.7408±0.0533 |
| Ada + PCA-40 | 0.6080±0.1180 | 0.5235±0.1911 | 0.4690±0.2162 | 0.4914±0.2054 | 0.6128±0.1416 |
| RF + PCA-50 | 0.6240±0.0921 | 0.6333±0.2828 | 0.3658±0.1187 | 0.4528±0.1550 | 0.5750±0.1155 |
| MLP + PCA-50 | 0.6480±0.0438 | 0.6253±0.0965 | 0.5469±0.1475 | 0.5639±0.0614 | 0.6844±0.1209 |
| KNN + PCA-50 | 0.5520±0.0769 | 0.4889±0.2056 | 0.2854±0.1444 | 0.3387±0.1612 | 0.6028±0.0979 |
| SVM + PCA-50 | 0.6480±0.0867 | 0.7633±0.2518 | 0.3849±0.1666 | 0.4732±0.1120 | 0.6564±0.1210 |
| LR + PCA-50 | 0.6160±0.0537 | 0.5986±0.1663 | 0.5891±0.1563 | 0.5618±0.0450 | 0.7129±0.0991 |
| DT + PCA-50 | 0.5200±0.1265 | 0.4667±0.1247 | 0.5299±0.0835 | 0.4902±0.0979 | 0.5188±0.1057 |
| GBDT + PCA-50 | 0.5280±0.0657 | 0.4505±0.1633 | 0.4068±0.1304 | 0.4202±0.1252 | 0.5100±0.1608 |
| LDA + PCA-50 | 0.6400±0.0400 | 0.6032±0.1324 | 0.5999±0.1463 | 0.5820±0.0677 | 0.6999±0.1182 |
| Ada + PCA-50 | 0.6160±0.1345 | 0.5833±0.2811 | 0.4755±0.1569 | 0.5140±0.1915 | 0.5716±0.1833 |
| RF + PCA-60 | 0.6160±0.0727 | 0.6776±0.3091 | 0.3703±0.0786 | 0.4530±0.0872 | 0.5937±0.1607 |
| MLP + PCA-60 | 0.6160±0.0358 | 0.5590±0.1177 | 0.5210±0.1388 | 0.5300±0.1015 | 0.6397±0.1278 |
| KNN + PCA-60 | 0.5680±0.0522 | 0.6000±0.5477 | 0.0828±0.0947 | 0.1421±0.1552 | 0.5613±0.1815 |
| SVM + PCA-60 | 0.5840±0.0669 | 0.6667±0.3118 | 0.2697±0.1119 | 0.3478±0.0855 | 0.5033±0.1823 |
| LR + PCA-60 | 0.6080±0.0657 | 0.5580±0.1166 | 0.5858±0.1512 | 0.5555±0.0853 | 0.6733±0.0806 |
| DT + PCA-60 | 0.5120±0.1110 | 0.4599±0.1461 | 0.4223±0.1183 | 0.4247±0.0740 | 0.4974±0.0925 |
| GBDT + PCA-60 | 0.5920±0.0820 | 0.5258±0.1577 | 0.4610±0.1594 | 0.4853±0.1397 | 0.5866±0.2126 |
| LDA + PCA-60 | 0.6000±0.0748 | 0.5414±0.1275 | 0.6221±0.1838 | 0.5645±0.1026 | 0.6708±0.1030 |
| Ada + PCA-60 | 0.6240±0.1004 | 0.5741±0.1965 | 0.5121±0.1338 | 0.5349±0.1514 | 0.6286±0.1131 |
| RF + ISOMAP-10 | 0.6200±0.1058 | 0.6396±0.2916 | 0.4429±0.2208 | 0.4963±0.1868 | 0.6504±0.1585 |
| MLP + ISOMAP-10 | 0.6500±0.0683 | 0.6275±0.1219 | 0.6080±0.2110 | 0.5985±0.1015 | 0.6512±0.1102 |
| KNN + ISOMAP-10 | 0.6000±0.0864 | 0.6250±0.1984 | 0.3529±0.0956 | 0.4406±0.1162 | 0.6471±0.1651 |
| SVM + ISOMAP-10 | 0.5600±0.0864 | 0.6292±0.2757 | 0.3285±0.2636 | 0.3598±0.2013 | 0.6767±0.1179 |
| LR + ISOMAP-10 | 0.5900±0.0600 | 0.6208±0.2638 | 0.3987±0.0970 | 0.4627±0.0755 | 0.6496±0.1082 |
| DT + ISOMAP-10 | 0.6000±0.0730 | 0.5662±0.1182 | 0.4888±0.1712 | 0.5135±0.1178 | 0.5877±0.0832 |
| GBDT + ISOMAP-10 | 0.6200±0.0766 | 0.6049±0.1699 | 0.4429±0.1486 | 0.5035±0.1407 | 0.6389±0.1555 |
| LDA + ISOMAP-10 | 0.5900±0.0683 | 0.6250±0.2700 | 0.4045±0.1349 | 0.4637±0.0916 | 0.6524±0.1137 |
| Ada + ISOMAP-10 | 0.5500±0.0825 | 0.5185±0.1902 | 0.3260±0.2524 | 0.3636±0.1762 | 0.6197±0.0893 |
| RF + ISOMAP-20 | 0.5200±0.0894 | 0.4423±0.2384 | 0.3126±0.1847 | 0.3453±0.1637 | 0.5444±0.1659 |
| MLP + ISOMAP-20 | 0.5600±0.0632 | 0.4692±0.1401 | 0.4827±0.2025 | 0.4685±0.1661 | 0.5352±0.1085 |
| KNN + ISOMAP-20 | 0.5520±0.0657 | 0.5591±0.2969 | 0.2685±0.1872 | 0.3185±0.1426 | 0.5467±0.1493 |
| SVM + ISOMAP-20 | 0.5520±0.0593 | 0.5466±0.2719 | 0.3382±0.1552 | 0.3808±0.0973 | 0.3933±0.1683 |
| LR + ISOMAP-20 | 0.5920±0.0867 | 0.5257±0.1387 | 0.4377±0.1916 | 0.4655±0.1652 | 0.5981±0.0985 |
| DT + ISOMAP-20 | 0.5280±0.0867 | 0.4200±0.1459 | 0.4355±0.2474 | 0.4221±0.1893 | 0.5107±0.1124 |
| GBDT + ISOMAP-20 | 0.5360±0.1403 | 0.4506±0.2461 | 0.3788±0.2413 | 0.3976±0.2272 | 0.5197±0.1874 |
| LDA + ISOMAP-20 | 0.5760±0.0727 | 0.5111±0.1250 | 0.4023±0.1487 | 0.4399±0.1349 | 0.5952±0.1122 |
| Ada + ISOMAP-20 | 0.5600±0.1020 | 0.4829±0.1855 | 0.4753±0.1938 | 0.4701±0.1763 | 0.5573±0.1202 |
| RF + ISOMAP-30 | 0.5840±0.0669 | 0.4898±0.3087 | 0.3580±0.2440 | 0.3859±0.2305 | 0.5167±0.1552 |
| MLP + ISOMAP-30 | 0.5920±0.1110 | 0.4654±0.2967 | 0.4553±0.2972 | 0.4519±0.2790 | 0.6085±0.1433 |
| KNN + ISOMAP-30 | 0.5280±0.0657 | 0.3778±0.2434 | 0.2471±0.1961 | 0.2840±0.2041 | 0.5081±0.1339 |
| SVM + ISOMAP-30 | 0.5920±0.0335 | 0.6410±0.2615 | 0.2226±0.1352 | 0.3004±0.1502 | 0.5156±0.2039 |
| LR + ISOMAP-30 | 0.6080±0.0522 | 0.5327±0.0887 | 0.5244±0.2309 | 0.5129±0.1533 | 0.6311±0.1344 |
| DT + ISOMAP-30 | 0.4480±0.1559 | 0.3681±0.1474 | 0.4336±0.2653 | 0.3900±0.1901 | 0.4492±0.1716 |
| GBDT + ISOMAP-30 | 0.4800±0.1166 | 0.3633±0.1743 | 0.2662±0.1565 | 0.2975±0.1585 | 0.5209±0.1186 |
| LDA + ISOMAP-30 | 0.6080±0.0716 | 0.5342±0.0874 | 0.5090±0.2323 | 0.5063±0.1571 | 0.6257±0.1344 |
| Ada + ISOMAP-30 | 0.5360±0.0607 | 0.4585±0.1397 | 0.4126±0.1516 | 0.4240±0.1240 | 0.4971±0.1347 |
| RF + ISOMAP-40 | 0.5920±0.1213 | 0.6000±0.3084 | 0.3263±0.2061 | 0.3881±0.2058 | 0.6548±0.1939 |
| MLP + ISOMAP-40 | 0.5920±0.0996 | 0.5271±0.1060 | 0.5256±0.1628 | 0.5197±0.1243 | 0.6733±0.1051 |
| KNN + ISOMAP-40 | 0.5360±0.0456 | 0.4444±0.0981 | 0.3017±0.1385 | 0.3425±0.1128 | 0.5479±0.0834 |
| SVM + ISOMAP-40 | 0.5840±0.1252 | 0.4714±0.3530 | 0.2439±0.2196 | 0.3088±0.2568 | 0.6460±0.1037 |
| LR + ISOMAP-40 | 0.6000±0.1265 | 0.5312±0.1205 | 0.4747±0.2067 | 0.4953±0.1636 | 0.6562±0.1201 |
| DT + ISOMAP-40 | 0.5360±0.1081 | 0.4765±0.1742 | 0.4686±0.2440 | 0.4466±0.1680 | 0.5348±0.1155 |
| GBDT + ISOMAP-40 | 0.5440±0.1590 | 0.5150±0.2255 | 0.4062±0.1761 | 0.4327±0.1423 | 0.5950±0.1390 |
| LDA + ISOMAP-40 | 0.6240±0.1513 | 0.5992±0.1741 | 0.4914±0.1351 | 0.5347±0.1417 | 0.6797±0.1110 |
| Ada + ISOMAP-40 | 0.6080±0.0955 | 0.5885±0.1207 | 0.4347±0.1350 | 0.4832±0.0887 | 0.6259±0.1118 |
| RF + ISOMAP-50 | 0.5760±0.0921 | 0.4962±0.3368 | 0.2615±0.1881 | 0.3230±0.2195 | 0.6440±0.1441 |
| MLP + ISOMAP-50 | 0.5680±0.1180 | 0.4943±0.1369 | 0.3971±0.1903 | 0.4303±0.1620 | 0.5825±0.1549 |
| KNN + ISOMAP-50 | 0.5680±0.0955 | 0.5500±0.3708 | 0.1862±0.1213 | 0.2673±0.1589 | 0.5719±0.1565 |
| SVM + ISOMAP-50 | 0.5840±0.0876 | 0.4267±0.4585 | 0.1578±0.1678 | 0.2171±0.2244 | 0.4834±0.1370 |
| LR + ISOMAP-50 | 0.5280±0.1308 | 0.4429±0.1502 | 0.4504±0.2279 | 0.4372±0.1815 | 0.5911±0.1449 |
| DT + ISOMAP-50 | 0.5600±0.1166 | 0.5438±0.2760 | 0.5215±0.1854 | 0.5000±0.1384 | 0.5516±0.1105 |
| GBDT + ISOMAP-50 | 0.5440±0.1711 | 0.5231±0.2832 | 0.3839±0.1844 | 0.4202±0.1880 | 0.5902±0.1497 |
| LDA + ISOMAP-50 | 0.5600±0.1356 | 0.4758±0.1558 | 0.4338±0.2264 | 0.4455±0.1902 | 0.6224±0.1341 |
| Ada + ISOMAP-50 | 0.5680±0.0769 | 0.4842±0.1793 | 0.3793±0.2031 | 0.4109±0.1739 | 0.6125±0.1352 |
| RF + ISOMAP-60 | 0.5600±0.0632 | 0.5000±0.3742 | 0.2062±0.1320 | 0.2670±0.1550 | 0.6148±0.0856 |
| MLP + ISOMAP-60 | 0.5680±0.1110 | 0.4931±0.1033 | 0.3885±0.1861 | 0.4220±0.1531 | 0.5876±0.0507 |
| KNN + ISOMAP-60 | 0.5360±0.1043 | 0.3611±0.4240 | 0.1432±0.2340 | 0.1647±0.2286 | 0.5684±0.0620 |
| SVM + ISOMAP-60 | 0.5680±0.0335 | 0.5857±0.4238 | 0.1187±0.1260 | 0.1677±0.1341 | 0.5880±0.0406 |
| LR + ISOMAP-60 | 0.6240±0.0876 | 0.5606±0.1096 | 0.5588±0.1477 | 0.5565±0.1177 | 0.6228±0.0839 |
| DT + ISOMAP-60 | 0.5200±0.0938 | 0.4760±0.2197 | 0.4444±0.1587 | 0.4380±0.1221 | 0.5153±0.0974 |
| GBDT + ISOMAP-60 | 0.5200±0.1265 | 0.4050±0.2502 | 0.3674±0.2858 | 0.3644±0.2476 | 0.5295±0.0958 |
| LDA + ISOMAP-60 | 0.6080±0.0657 | 0.5466±0.0306 | 0.5447±0.1155 | 0.5407±0.0659 | 0.5918±0.0784 |
| Ada + ISOMAP-60 | 0.6160±0.0456 | 0.6136±0.1518 | 0.4569±0.1319 | 0.4985±0.0433 | 0.6125±0.0283 |
| RF + UMAP-10 | 0.6240±0.1187 | 0.5803±0.1995 | 0.5496±0.1093 | 0.5571±0.1393 | 0.6447±0.1476 |
| MLP + UMAP-10 | 0.6160±0.2012 | 0.5917±0.2472 | 0.5477±0.1908 | 0.5594±0.1940 | 0.6363±0.1515 |
| KNN + UMAP-10 | 0.6000±0.1575 | 0.6037±0.2694 | 0.4356±0.2068 | 0.4796±0.1956 | 0.6504±0.1924 |
| SVM + UMAP-10 | 0.5120±0.1775 | 0.4248±0.3022 | 0.2473±0.2085 | 0.3000±0.2437 | 0.3913±0.1825 |
| LR + UMAP-10 | 0.5840±0.1081 | 0.5655±0.2145 | 0.4182±0.1791 | 0.4548±0.1356 | 0.6102±0.1574 |
| DT + UMAP-10 | 0.6400±0.1095 | 0.5699±0.1835 | 0.6285±0.2175 | 0.5880±0.1732 | 0.6430±0.1264 |
| GBDT + UMAP-10 | 0.6000±0.1166 | 0.5596±0.2101 | 0.5986±0.1515 | 0.5610±0.1315 | 0.6746±0.1460 |
| LDA + UMAP-10 | 0.5600±0.1200 | 0.5226±0.2343 | 0.3515±0.1135 | 0.4094±0.1445 | 0.5862±0.1700 |
| Ada + UMAP-10 | 0.6080±0.1035 | 0.6361±0.2871 | 0.5264±0.1937 | 0.5227±0.1405 | 0.6794±0.1234 |
| RF + UMAP-20 | 0.6240±0.1043 | 0.6043±0.2029 | 0.5666±0.1684 | 0.5601±0.1147 | 0.6857±0.1744 |
| MLP + UMAP-20 | 0.5360±0.1043 | 0.4861±0.1856 | 0.5010±0.1316 | 0.4796±0.1204 | 0.5791±0.1561 |
| KNN + UMAP-20 | 0.5920±0.1397 | 0.5872±0.2674 | 0.3632±0.1312 | 0.4368±0.1651 | 0.6539±0.1756 |
| SVM + UMAP-20 | 0.5120±0.0955 | 0.5333±0.3116 | 0.2473±0.1161 | 0.2931±0.1200 | 0.4685±0.1953 |
| LR + UMAP-20 | 0.5040±0.1345 | 0.4128±0.2480 | 0.2897±0.1849 | 0.3293±0.2068 | 0.6389±0.1731 |
| DT + UMAP-20 | 0.6160±0.1431 | 0.5369±0.1837 | 0.5775±0.2457 | 0.5534±0.2060 | 0.6120±0.1618 |
| GBDT + UMAP-20 | 0.6800±0.1697 | 0.6230±0.2426 | 0.6396±0.2265 | 0.6254±0.2180 | 0.7284±0.1545 |
| LDA + UMAP-20 | 0.5040±0.1081 | 0.4308±0.2389 | 0.2717±0.1119 | 0.3247±0.1481 | 0.6219±0.1698 |
| Ada + UMAP-20 | 0.6400±0.1020 | 0.6152±0.2039 | 0.5977±0.0844 | 0.5903±0.0988 | 0.7039±0.1744 |
| RF + UMAP-30 | 0.5920±0.1180 | 0.5709±0.2322 | 0.5444±0.1977 | 0.5283±0.1390 | 0.6192±0.1666 |
| MLP + UMAP-30 | 0.5360±0.1220 | 0.4910±0.1596 | 0.4455±0.0968 | 0.4549±0.1019 | 0.5296±0.1407 |
| KNN + UMAP-30 | 0.6160±0.1590 | 0.6043±0.2606 | 0.4690±0.1812 | 0.5150±0.1885 | 0.6583±0.1714 |
| SVM + UMAP-30 | 0.5360±0.1081 | 0.5879±0.2943 | 0.2930±0.1321 | 0.3405±0.1016 | 0.5338±0.2007 |
| LR + UMAP-30 | 0.6080±0.1339 | 0.6155±0.2644 | 0.4258±0.0850 | 0.4906±0.1392 | 0.6827±0.1541 |
| DT + UMAP-30 | 0.5440±0.1284 | 0.4889±0.2581 | 0.4310±0.1249 | 0.4510±0.1733 | 0.5327±0.1410 |
| GBDT + UMAP-30 | 0.5600±0.1497 | 0.5169±0.2212 | 0.5164±0.1412 | 0.5055±0.1592 | 0.5875±0.1559 |
| LDA + UMAP-30 | 0.6240±0.1664 | 0.6051±0.2699 | 0.4945±0.1433 | 0.5371±0.1940 | 0.6822±0.1735 |
| Ada + UMAP-30 | 0.5760±0.1565 | 0.5100±0.2135 | 0.5174±0.2289 | 0.5046±0.2020 | 0.5932±0.1193 |
| RF + UMAP-40 | 0.6320±0.0955 | 0.6013±0.2137 | 0.5632±0.1862 | 0.5607±0.1387 | 0.6647±0.1209 |
| MLP + UMAP-40 | 0.6080±0.0955 | 0.5459±0.1275 | 0.5266±0.2302 | 0.5180±0.1633 | 0.6186±0.1275 |
| KNN + UMAP-40 | 0.6000±0.1020 | 0.5504±0.2432 | 0.3901±0.1816 | 0.4458±0.1884 | 0.6399±0.1658 |
| SVM + UMAP-40 | 0.5200±0.1296 | 0.4476±0.1931 | 0.3049±0.1924 | 0.3380±0.1921 | 0.5431±0.2095 |
| LR + UMAP-40 | 0.5600±0.1356 | 0.5150±0.2395 | 0.3341±0.1557 | 0.3902±0.1829 | 0.6517±0.1662 |
| DT + UMAP-40 | 0.4640±0.1345 | 0.3873±0.1851 | 0.4118±0.1610 | 0.3967±0.1728 | 0.4568±0.1368 |
| GBDT + UMAP-40 | 0.5840±0.1459 | 0.5283±0.1804 | 0.5185±0.1588 | 0.5151±0.1570 | 0.5864±0.1279 |
| LDA + UMAP-40 | 0.5360±0.1220 | 0.4759±0.2459 | 0.3106±0.1121 | 0.3689±0.1566 | 0.6436±0.1810 |
| Ada + UMAP-40 | 0.5760±0.1187 | 0.5356±0.1765 | 0.4642±0.1436 | 0.4792±0.1366 | 0.6135±0.1506 |
| RF + UMAP-50 | 0.6640±0.1284 | 0.6245±0.1950 | 0.5786±0.1868 | 0.5925±0.1683 | 0.6915±0.1509 |
| MLP + UMAP-50 | 0.5920±0.1559 | 0.5715±0.2336 | 0.4925±0.1804 | 0.5110±0.1669 | 0.6057±0.1598 |
| KNN + UMAP-50 | 0.6000±0.1625 | 0.5803±0.2740 | 0.4232±0.1604 | 0.4815±0.1991 | 0.6643±0.1989 |
| SVM + UMAP-50 | 0.5920±0.1585 | 0.5789±0.2588 | 0.4334±0.2091 | 0.4719±0.2021 | 0.6645±0.1860 |
| LR + UMAP-50 | 0.6640±0.1403 | 0.6376±0.2158 | 0.5625±0.2142 | 0.5818±0.1851 | 0.7383±0.1721 |
| DT + UMAP-50 | 0.6240±0.1004 | 0.5706±0.1603 | 0.5388±0.1782 | 0.5458±0.1397 | 0.6155±0.1090 |
| GBDT + UMAP-50 | 0.6240±0.1220 | 0.6123±0.2350 | 0.4899±0.1910 | 0.5215±0.1599 | 0.6751±0.1400 |
| LDA + UMAP-50 | 0.6400±0.1296 | 0.6103±0.2245 | 0.5403±0.1907 | 0.5561±0.1732 | 0.7303±0.1736 |
| Ada + UMAP-50 | 0.7200±0.0894 | 0.6930±0.1795 | 0.6516±0.1992 | 0.6546±0.1434 | 0.6735±0.1145 |
| RF + UMAP-60 | 0.6320±0.0867 | 0.6098±0.1848 | 0.5197±0.1184 | 0.5460±0.1032 | 0.6433±0.1469 |
| MLP + UMAP-60 | 0.5440±0.0607 | 0.4731±0.0961 | 0.4888±0.1382 | 0.4711±0.1018 | 0.5524±0.0849 |
| KNN + UMAP-60 | 0.6000±0.1233 | 0.5619±0.2617 | 0.3480±0.1603 | 0.4271±0.1946 | 0.6191±0.1737 |
| SVM + UMAP-60 | 0.4880±0.1035 | 0.3897±0.2009 | 0.1617±0.0562 | 0.2148±0.0788 | 0.4029±0.1620 |
| LR + UMAP-60 | 0.6320±0.1073 | 0.6289±0.2389 | 0.4812±0.0755 | 0.5330±0.1220 | 0.6713±0.1251 |
| DT + UMAP-60 | 0.5920±0.0657 | 0.5178±0.1444 | 0.5262±0.1445 | 0.5177±0.1357 | 0.5813±0.0763 |
| GBDT + UMAP-60 | 0.6080±0.0996 | 0.5566±0.1738 | 0.5696±0.1283 | 0.5527±0.1284 | 0.6066±0.1357 |
| LDA + UMAP-60 | 0.6160±0.1315 | 0.5908±0.2306 | 0.5166±0.1153 | 0.5404±0.1494 | 0.6432±0.1320 |
| Ada + UMAP-60 | 0.5920±0.0996 | 0.5306±0.1665 | 0.5155±0.1228 | 0.5192±0.1363 | 0.6070±0.1497 |
| RF + SPECTRAL-10 | 0.6160±0.1117 | 0.5844±0.2051 | 0.5210±0.0962 | 0.5410±0.1204 | 0.6326±0.1094 |
| MLP + SPECTRAL-10 | 0.5840±0.0780 | 0.5200±0.1609 | 0.4788±0.1599 | 0.4873±0.1412 | 0.5700±0.1192 |
| KNN + SPECTRAL-10 | 0.6000±0.0894 | 0.5706±0.1787 | 0.4445±0.0391 | 0.4923±0.0908 | 0.6181±0.1683 |
| SVM + SPECTRAL-10 | 0.6160±0.1252 | 0.5956±0.2168 | 0.5312±0.2004 | 0.5376±0.1472 | 0.6179±0.1608 |
| LR + SPECTRAL-10 | 0.6480±0.1683 | 0.6079±0.2395 | 0.5877±0.1949 | 0.5879±0.1942 | 0.6695±0.1463 |
| DT + SPECTRAL-10 | 0.6240±0.1345 | 0.5832±0.2062 | 0.5321±0.1389 | 0.5510±0.1578 | 0.6130±0.1355 |
| GBDT + SPECTRAL-10 | 0.5840±0.1374 | 0.5336±0.2315 | 0.4610±0.1594 | 0.4878±0.1721 | 0.5739±0.1211 |
| LDA + SPECTRAL-10 | 0.6400±0.1470 | 0.5891±0.1943 | 0.6077±0.1600 | 0.5911±0.1623 | 0.6639±0.1449 |
| Ada + SPECTRAL-10 | 0.6400±0.1327 | 0.6119±0.2599 | 0.5510±0.1723 | 0.5659±0.1725 | 0.6451±0.1374 |
| RF + SPECTRAL-20 | 0.6240±0.0963 | 0.5806±0.1849 | 0.5232±0.1702 | 0.5382±0.1405 | 0.6204±0.1018 |
| MLP + SPECTRAL-20 | 0.5920±0.0912 | 0.5232±0.1840 | 0.4009±0.1768 | 0.4465±0.1703 | 0.6272±0.1534 |
| KNN + SPECTRAL-20 | 0.6080±0.0769 | 0.6410±0.2615 | 0.3349±0.0394 | 0.4262±0.0718 | 0.6152±0.1533 |
| SVM + SPECTRAL-20 | 0.6480±0.0912 | 0.6628±0.2362 | 0.5299±0.1746 | 0.5564±0.1170 | 0.6315±0.1065 |
| LR + SPECTRAL-20 | 0.6320±0.0955 | 0.5926±0.1523 | 0.5699±0.2133 | 0.5590±0.1360 | 0.6635±0.1272 |
| DT + SPECTRAL-20 | 0.5200±0.0800 | 0.4280±0.1514 | 0.3812±0.1265 | 0.4024±0.1367 | 0.5002±0.0881 |
| GBDT + SPECTRAL-20 | 0.6160±0.1152 | 0.5913±0.2159 | 0.4866±0.1835 | 0.5150±0.1488 | 0.5842±0.1187 |
| LDA + SPECTRAL-20 | 0.6240±0.0921 | 0.5860±0.1541 | 0.5499±0.1897 | 0.5474±0.1267 | 0.6753±0.1294 |
| Ada + SPECTRAL-20 | 0.6400±0.1131 | 0.5877±0.1595 | 0.5220±0.1837 | 0.5476±0.1627 | 0.6307±0.1315 |
| RF + SPECTRAL-30 | 0.6560±0.0829 | 0.6308±0.1765 | 0.5066±0.1770 | 0.5492±0.1396 | 0.6276±0.1424 |
| MLP + SPECTRAL-30 | 0.5680±0.1073 | 0.5161±0.1642 | 0.4712±0.1813 | 0.4769±0.1288 | 0.5915±0.1373 |
| KNN + SPECTRAL-30 | 0.5760±0.1252 | 0.5600±0.3343 | 0.2604±0.1625 | 0.3437±0.1953 | 0.6368±0.1681 |
| SVM + SPECTRAL-30 | 0.6000±0.1233 | 0.5859±0.2451 | 0.4356±0.2068 | 0.4725±0.1768 | 0.6181±0.1314 |
| LR + SPECTRAL-30 | 0.6000±0.1131 | 0.5523±0.1379 | 0.5312±0.1587 | 0.5309±0.1204 | 0.6090±0.1358 |
| DT + SPECTRAL-30 | 0.5040±0.1345 | 0.4124±0.1834 | 0.4231±0.2057 | 0.4163±0.1926 | 0.4858±0.1382 |
| GBDT + SPECTRAL-30 | 0.6320±0.0996 | 0.5725±0.1357 | 0.5351±0.1700 | 0.5475±0.1480 | 0.6008±0.1286 |
| LDA + SPECTRAL-30 | 0.6080±0.1145 | 0.5589±0.1455 | 0.5666±0.1684 | 0.5511±0.1268 | 0.6481±0.1165 |
| Ada + SPECTRAL-30 | 0.5840±0.1374 | 0.5114±0.1832 | 0.4866±0.2090 | 0.4940±0.1898 | 0.6047±0.1463 |
| RF + SPECTRAL-40 | 0.6400±0.0748 | 0.6469±0.2574 | 0.4442±0.2021 | 0.4945±0.1724 | 0.6232±0.1442 |
| MLP + SPECTRAL-40 | 0.5440±0.0669 | 0.4679±0.1484 | 0.4523±0.1908 | 0.4470±0.1394 | 0.5502±0.1077 |
| KNN + SPECTRAL-40 | 0.5600±0.0800 | 0.5242±0.1436 | 0.3485±0.1466 | 0.3932±0.1111 | 0.5953±0.1596 |
| SVM + SPECTRAL-40 | 0.5360±0.1081 | 0.4850±0.2497 | 0.3226±0.1698 | 0.3670±0.1566 | 0.5887±0.1234 |
| LR + SPECTRAL-40 | 0.5680±0.1073 | 0.5042±0.1403 | 0.4910±0.1668 | 0.4874±0.1382 | 0.5533±0.1609 |
| DT + SPECTRAL-40 | 0.5040±0.1187 | 0.4328±0.1481 | 0.4345±0.1508 | 0.4281±0.1339 | 0.4907±0.1117 |
| GBDT + SPECTRAL-40 | 0.5520±0.0912 | 0.5177±0.2016 | 0.3947±0.0984 | 0.4308±0.0899 | 0.5779±0.1134 |
| LDA + SPECTRAL-40 | 0.5600±0.0938 | 0.4982±0.1300 | 0.5277±0.1606 | 0.5018±0.1136 | 0.5732±0.1480 |
| Ada + SPECTRAL-40 | 0.5760±0.0727 | 0.5091±0.1562 | 0.4503±0.1723 | 0.4666±0.1374 | 0.5509±0.0697 |
| RF + SPECTRAL-50 | 0.5760±0.1004 | 0.5778±0.2849 | 0.3712±0.1361 | 0.4253±0.1312 | 0.5724±0.1736 |
| MLP + SPECTRAL-50 | 0.5200±0.0632 | 0.4256±0.1375 | 0.4056±0.1888 | 0.4083±0.1453 | 0.5223±0.0633 |
| KNN + SPECTRAL-50 | 0.5520±0.1073 | 0.5378±0.2796 | 0.2726±0.0581 | 0.3524±0.1190 | 0.5718±0.1668 |
| SVM + SPECTRAL-50 | 0.5360±0.0727 | 0.4350±0.2363 | 0.2649±0.1746 | 0.3111±0.1742 | 0.6014±0.0780 |
| LR + SPECTRAL-50 | 0.5200±0.1356 | 0.4381±0.1751 | 0.4310±0.1987 | 0.4284±0.1798 | 0.5338±0.1293 |
| DT + SPECTRAL-50 | 0.5040±0.1459 | 0.4188±0.1662 | 0.3847±0.1850 | 0.3964±0.1740 | 0.4862±0.1431 |
| GBDT + SPECTRAL-50 | 0.5600±0.0849 | 0.4806±0.1210 | 0.4173±0.1880 | 0.4348±0.1486 | 0.5564±0.0996 |
| LDA + SPECTRAL-50 | 0.5440±0.0921 | 0.4719±0.1200 | 0.4877±0.1630 | 0.4720±0.1240 | 0.5564±0.1086 |
| Ada + SPECTRAL-50 | 0.5520±0.1180 | 0.4891±0.1636 | 0.4656±0.1354 | 0.4725±0.1366 | 0.5384±0.1157 |
| RF + SPECTRAL-60 | 0.6000±0.1095 | 0.4495±0.3480 | 0.2680±0.2221 | 0.3319±0.2623 | 0.5133±0.1389 |
| MLP + SPECTRAL-60 | 0.4800±0.0938 | 0.3693±0.2162 | 0.3868±0.2221 | 0.3731±0.2100 | 0.5056±0.1044 |
| KNN + SPECTRAL-60 | 0.5440±0.0727 | 0.4705±0.1408 | 0.2352±0.1827 | 0.2827±0.1400 | 0.5393±0.0681 |
| SVM + SPECTRAL-60 | 0.5200±0.1020 | 0.4190±0.2501 | 0.1741±0.1416 | 0.2292±0.1619 | 0.4829±0.0617 |
| LR + SPECTRAL-60 | 0.4960±0.0669 | 0.4019±0.1089 | 0.3934±0.1583 | 0.3914±0.1249 | 0.5055±0.1133 |
| DT + SPECTRAL-60 | 0.5040±0.0358 | 0.4272±0.0918 | 0.4480±0.1604 | 0.4243±0.1039 | 0.5010±0.0343 |
| GBDT + SPECTRAL-60 | 0.5280±0.1073 | 0.4430±0.1732 | 0.3244±0.1299 | 0.3700±0.1402 | 0.5465±0.1199 |
| LDA + SPECTRAL-60 | 0.5040±0.1081 | 0.4043±0.1671 | 0.4090±0.2030 | 0.4032±0.1805 | 0.5083±0.1488 |
| Ada + SPECTRAL-60 | 0.5600±0.0490 | 0.4766±0.1497 | 0.3623±0.1017 | 0.4109±0.1201 | 0.5370±0.1180 |

**Table A3.** The performance of the preoperative prediction model in Experiment 3.

| **Predictor + Features** | **Evaluation metrics** | | | | |
| --- | --- | --- | --- | --- | --- |
|  | Mean accuracy ± SD | Mean precision ± SD | Mean recall ± SD | Mean F1-score ± SD | Mean AUC ± SD |
| RF + LASSO + ICA-10 | 0.7280±0.0996 | 0.7021±0.2145 | 0.6886±0.1904 | 0.6761±0.1485 | 0.8070±0.1518 |
| MLP + LASSO + ICA-10 | 0.6800±0.0938 | 0.6511±0.2115 | 0.6285±0.0854 | 0.6271±0.1138 | 0.7609±0.1137 |
| KNN + LASSO + ICA-10 | 0.6800±0.0748 | 0.6755±0.1674 | 0.4768±0.1411 | 0.5537±0.1384 | 0.7371±0.1241 |
| SVM + LASSO + ICA-10 | 0.6880±0.0522 | 0.6411±0.1285 | 0.6264±0.0918 | 0.6287±0.0919 | 0.7481±0.1372 |
| LR + LASSO + ICA-10 | 0.6720±0.0769 | 0.6511±0.2115 | 0.6131±0.0776 | 0.6159±0.0906 | 0.7490±0.1038 |
| DT + LASSO + ICA-10 | 0.6320±0.1073 | 0.5948±0.2453 | 0.4390±0.1875 | 0.4967±0.1938 | 0.6116±0.1299 |
| GBDT + LASSO + ICA-10 | 0.6960±0.0669 | 0.7089±0.2214 | 0.5345±0.0954 | 0.5981±0.1111 | 0.7788±0.1559 |
| LDA + LASSO + ICA-10 | 0.6800±0.0748 | 0.6401±0.1754 | 0.5475±0.1439 | 0.5863±0.1478 | 0.7639±0.1150 |
| Ada + LASSO + ICA-10 | 0.6960±0.0963 | 0.6914±0.2269 | 0.6134±0.1523 | 0.6288±0.1296 | 0.7816±0.1287 |
| RF + LASSO + ICA-20 | 0.6560±0.0727 | 0.6344±0.1706 | 0.5858±0.1512 | 0.5875±0.0979 | 0.7366±0.1264 |
| MLP + LASSO + ICA-20 | 0.6640±0.0727 | 0.6081±0.1558 | 0.6651±0.1075 | 0.6262±0.1034 | 0.7528±0.1121 |
| KNN + LASSO + ICA-20 | 0.6640±0.0669 | 0.6603±0.1990 | 0.4779±0.0690 | 0.5489±0.1088 | 0.7370±0.1179 |
| SVM + LASSO + ICA-20 | 0.6480±0.0912 | 0.6097±0.1958 | 0.6088±0.0835 | 0.5972±0.1109 | 0.7484±0.1385 |
| LR + LASSO + ICA-20 | 0.6640±0.0537 | 0.6155±0.1419 | 0.6497±0.1081 | 0.6206±0.0809 | 0.7690±0.0789 |
| DT + LASSO + ICA-20 | 0.6400±0.0800 | 0.5965±0.1388 | 0.5345±0.0954 | 0.5593±0.1070 | 0.6288±0.0807 |
| GBDT + LASSO + ICA-20 | 0.6400±0.0849 | 0.6161±0.1714 | 0.4825±0.0738 | 0.5358±0.1051 | 0.7172±0.1552 |
| LDA + LASSO + ICA-20 | 0.6880±0.0335 | 0.6377±0.1017 | 0.6720±0.0714 | 0.6475±0.0413 | 0.7787±0.0726 |
| Ada + LASSO + ICA-20 | 0.6880±0.0657 | 0.6542±0.1430 | 0.5868±0.1874 | 0.6046±0.1213 | 0.7148±0.0970 |
| RF + LASSO + ICA-30 | 0.6640±0.0876 | 0.6578±0.2433 | 0.5868±0.2247 | 0.5876±0.1430 | 0.7508±0.1394 |
| MLP + LASSO + ICA-30 | 0.7040±0.0727 | 0.6650±0.1756 | 0.7086±0.1544 | 0.6674±0.1010 | 0.7715±0.1060 |
| KNN + LASSO + ICA-30 | 0.6640±0.0537 | 0.7243±0.1975 | 0.4171±0.1156 | 0.5098±0.0877 | 0.7434±0.1553 |
| SVM + LASSO + ICA-30 | 0.6640±0.0780 | 0.6580±0.2347 | 0.5812±0.1529 | 0.5920±0.1116 | 0.7158±0.1420 |
| LR + LASSO + ICA-30 | 0.6960±0.0537 | 0.6508±0.1370 | 0.6953±0.1385 | 0.6588±0.0683 | 0.7709±0.0749 |
| DT + LASSO + ICA-30 | 0.5840±0.0727 | 0.4974±0.1151 | 0.5077±0.1849 | 0.5002±0.1492 | 0.5675±0.0887 |
| GBDT + LASSO + ICA-30 | 0.6640±0.0829 | 0.6728±0.2537 | 0.4979±0.0887 | 0.5594±0.1214 | 0.7132±0.1422 |
| LDA + LASSO + ICA-30 | 0.6640±0.0727 | 0.6083±0.1491 | 0.6285±0.1493 | 0.6095±0.1194 | 0.7602±0.0754 |
| Ada + LASSO + ICA-30 | 0.6320±0.1073 | 0.6210±0.2595 | 0.5145±0.1132 | 0.5458±0.1374 | 0.7135±0.1548 |
| RF + LASSO + ICA-40 | 0.6640±0.0358 | 0.6802±0.1949 | 0.5780±0.2455 | 0.5776±0.0900 | 0.7705±0.1355 |
| MLP + LASSO + ICA-40 | 0.7200±0.0283 | 0.6950±0.1409 | 0.7188±0.1667 | 0.6824±0.0403 | 0.7599±0.0385 |
| KNN + LASSO + ICA-40 | 0.6160±0.0456 | 0.7000±0.2739 | 0.3041±0.0915 | 0.3972±0.0871 | 0.7002±0.1512 |
| SVM + LASSO + ICA-40 | 0.6400±0.0748 | 0.6222±0.1785 | 0.5891±0.1563 | 0.5785±0.0868 | 0.7070±0.1184 |
| LR + LASSO + ICA-40 | 0.6960±0.0727 | 0.6502±0.1689 | 0.7286±0.1241 | 0.6704±0.0857 | 0.7713±0.0523 |
| DT + LASSO + ICA-40 | 0.6320±0.1453 | 0.5448±0.3519 | 0.5190±0.3143 | 0.5109±0.3011 | 0.6262±0.1684 |
| GBDT + LASSO + ICA-40 | 0.7120±0.0657 | 0.7535±0.2300 | 0.5847±0.1479 | 0.6293±0.0941 | 0.7543±0.1261 |
| LDA + LASSO + ICA-40 | 0.6880±0.0867 | 0.6387±0.1537 | 0.6955±0.1689 | 0.6515±0.1122 | 0.7403±0.0983 |
| Ada + LASSO + ICA-40 | 0.6480±0.0867 | 0.6333±0.2401 | 0.6134±0.1523 | 0.5946±0.1106 | 0.7143±0.1182 |
| RF + LASSO + ICA-50 | 0.6640±0.0358 | 0.6733±0.2064 | 0.5747±0.2160 | 0.5806±0.0867 | 0.7163±0.1518 |
| MLP + LASSO + ICA-50 | 0.6320±0.0522 | 0.6468±0.2307 | 0.5503±0.1308 | 0.5574±0.0344 | 0.7298±0.0950 |
| KNN + LASSO + ICA-50 | 0.6320±0.0179 | 0.6832±0.2182 | 0.3571±0.0627 | 0.4512±0.0308 | 0.7120±0.1653 |
| SVM + LASSO + ICA-50 | 0.6400±0.0980 | 0.6525±0.2420 | 0.5915±0.2178 | 0.5727±0.1225 | 0.6773±0.1430 |
| LR + LASSO + ICA-50 | 0.6720±0.0657 | 0.6621±0.2161 | 0.6379±0.1467 | 0.6200±0.0826 | 0.7282±0.0837 |
| DT + LASSO + ICA-50 | 0.6000±0.1356 | 0.4981±0.2347 | 0.4715±0.3295 | 0.4664±0.2638 | 0.5831±0.1598 |
| GBDT + LASSO + ICA-50 | 0.6480±0.1110 | 0.6964±0.2529 | 0.4950±0.2034 | 0.5341±0.1550 | 0.7243±0.1458 |
| LDA + LASSO + ICA-50 | 0.6240±0.0727 | 0.5691±0.1084 | 0.6058±0.1599 | 0.5735±0.0973 | 0.7089±0.1144 |
| Ada + LASSO + ICA-50 | 0.6320±0.1035 | 0.5958±0.1823 | 0.5826±0.2441 | 0.5580±0.1624 | 0.7301±0.1244 |
| RF + LASSO + ICA-60 | 0.6240±0.0456 | 0.6397±0.2272 | 0.4736±0.1168 | 0.5149±0.0416 | 0.7190±0.0654 |
| MLP + LASSO + ICA-60 | 0.5920±0.0593 | 0.5447±0.1142 | 0.5482±0.1508 | 0.5266±0.0902 | 0.6639±0.0830 |
| KNN + LASSO + ICA-60 | 0.5600±0.0400 | 0.5657±0.3035 | 0.1898±0.0970 | 0.2599±0.1118 | 0.6718±0.1570 |
| SVM + LASSO + ICA-60 | 0.5760±0.0727 | 0.5883±0.2635 | 0.4895±0.1979 | 0.4809±0.1204 | 0.6281±0.1134 |
| LR + LASSO + ICA-60 | 0.5840±0.0537 | 0.5273±0.0797 | 0.5891±0.1394 | 0.5433±0.0585 | 0.6891±0.0822 |
| DT + LASSO + ICA-60 | 0.6080±0.0769 | 0.5141±0.2017 | 0.5131±0.2330 | 0.5051±0.2128 | 0.5970±0.1039 |
| GBDT + LASSO + ICA-60 | 0.6400±0.0632 | 0.6537±0.2060 | 0.5349±0.1571 | 0.5520±0.0720 | 0.7196±0.1184 |
| LDA + LASSO + ICA-60 | 0.6000±0.1131 | 0.5379±0.1341 | 0.6025±0.1662 | 0.5591±0.1302 | 0.6663±0.1041 |
| Ada + LASSO + ICA-60 | 0.7040±0.0669 | 0.6847±0.1009 | 0.6671±0.2275 | 0.6435±0.1198 | 0.7137±0.0844 |
| RF + LASSO + PCA-10 | 0.7120±0.0955 | 0.6851±0.1991 | 0.6486±0.1521 | 0.6531±0.1400 | 0.7524±0.1648 |
| MLP + LASSO + PCA-10 | 0.6880±0.0867 | 0.6611±0.2155 | 0.6464±0.0954 | 0.6386±0.1093 | 0.7468±0.0915 |
| KNN + LASSO + PCA-10 | 0.6880±0.0820 | 0.6775±0.1487 | 0.4921±0.1432 | 0.5683±0.1451 | 0.7418±0.1282 |
| SVM + LASSO + PCA-10 | 0.6960±0.0669 | 0.6547±0.1454 | 0.6264±0.0918 | 0.6352±0.1031 | 0.7481±0.1406 |
| LR + LASSO + PCA-10 | 0.6720±0.0593 | 0.6602±0.2042 | 0.5977±0.0844 | 0.6092±0.0644 | 0.7452±0.0999 |
| DT + LASSO + PCA-10 | 0.6720±0.0769 | 0.6395±0.1699 | 0.5107±0.1778 | 0.5582±0.1571 | 0.6533±0.0975 |
| GBDT + LASSO + PCA-10 | 0.6800±0.0980 | 0.6547±0.1827 | 0.5758±0.1872 | 0.5967±0.1520 | 0.7509±0.1423 |
| LDA + LASSO + PCA-10 | 0.6880±0.0867 | 0.6623±0.2169 | 0.5475±0.1439 | 0.5932±0.1566 | 0.7614±0.1143 |
| Ada + LASSO + PCA-10 | 0.6880±0.1180 | 0.6683±0.2344 | 0.6468±0.2003 | 0.6308±0.1521 | 0.7520±0.1450 |
| RF + LASSO + PCA-20 | 0.7360±0.1004 | 0.6918±0.1745 | 0.7109±0.2090 | 0.6882±0.1519 | 0.7679±0.1210 |
| MLP + LASSO + PCA-20 | 0.6480±0.0769 | 0.6011±0.1844 | 0.6275±0.1150 | 0.6020±0.1090 | 0.7110±0.0871 |
| KNN + LASSO + PCA-20 | 0.6800±0.0748 | 0.6806±0.1718 | 0.5014±0.1164 | 0.5688±0.1172 | 0.6949±0.1439 |
| SVM + LASSO + PCA-20 | 0.6560±0.0669 | 0.6056±0.1484 | 0.5888±0.0970 | 0.5916±0.1052 | 0.7393±0.1319 |
| LR + LASSO + PCA-20 | 0.6640±0.0537 | 0.6186±0.1568 | 0.6497±0.1081 | 0.6210±0.0765 | 0.7466±0.1012 |
| DT + LASSO + PCA-20 | 0.6640±0.0607 | 0.6365±0.1761 | 0.5410±0.1588 | 0.5712±0.1252 | 0.6555±0.0806 |
| GBDT + LASSO + PCA-20 | 0.6800±0.1296 | 0.6505±0.2443 | 0.5853±0.1642 | 0.6075±0.1805 | 0.7103±0.1714 |
| LDA + LASSO + PCA-20 | 0.6800±0.0632 | 0.6355±0.1547 | 0.6320±0.1425 | 0.6227±0.1081 | 0.7640±0.1066 |
| Ada + LASSO + PCA-20 | 0.6480±0.1339 | 0.6160±0.2334 | 0.5853±0.1093 | 0.5909±0.1575 | 0.6843±0.1402 |
| RF + LASSO + PCA-30 | 0.7120±0.1110 | 0.7175±0.2679 | 0.6344±0.1849 | 0.6485±0.1530 | 0.7532±0.1436 |
| MLP + LASSO + PCA-30 | 0.6880±0.0593 | 0.6646±0.2069 | 0.6988±0.1890 | 0.6509±0.0854 | 0.7369±0.0938 |
| KNN + LASSO + PCA-30 | 0.6880±0.0716 | 0.7693±0.1825 | 0.4517±0.1507 | 0.5434±0.1247 | 0.7224±0.1574 |
| SVM + LASSO + PCA-30 | 0.6720±0.0657 | 0.6441±0.1795 | 0.5966±0.1412 | 0.6037±0.1068 | 0.7207±0.1232 |
| LR + LASSO + PCA-30 | 0.6880±0.0438 | 0.6501±0.1291 | 0.6599±0.1716 | 0.6377±0.0737 | 0.7498±0.1037 |
| DT + LASSO + PCA-30 | 0.6720±0.0867 | 0.6308±0.1720 | 0.5986±0.2073 | 0.5983±0.1497 | 0.6696±0.1035 |
| GBDT + LASSO + PCA-30 | 0.6640±0.0607 | 0.6246±0.1513 | 0.5521±0.0984 | 0.5812±0.1075 | 0.6993±0.1424 |
| LDA + LASSO + PCA-30 | 0.6560±0.0829 | 0.6133±0.1871 | 0.5999±0.2035 | 0.5886±0.1434 | 0.7606±0.1068 |
| Ada + LASSO + PCA-30 | 0.6720±0.1035 | 0.6361±0.1944 | 0.5479±0.1762 | 0.5793±0.1596 | 0.7109±0.1465 |
| RF + LASSO + PCA-40 | 0.7040±0.0829 | 0.7343±0.2453 | 0.5590±0.1221 | 0.6147±0.1199 | 0.7313±0.1499 |
| MLP + LASSO + PCA-40 | 0.6960±0.0607 | 0.6786±0.2020 | 0.6755±0.2008 | 0.6455±0.1060 | 0.7377±0.0972 |
| KNN + LASSO + PCA-40 | 0.6240±0.0780 | 0.6422±0.2568 | 0.3295±0.1515 | 0.4190±0.1532 | 0.6838±0.1770 |
| SVM + LASSO + PCA-40 | 0.6640±0.0669 | 0.6404±0.1785 | 0.5790±0.1377 | 0.5907±0.1052 | 0.7271±0.1352 |
| LR + LASSO + PCA-40 | 0.6720±0.0657 | 0.6188±0.1434 | 0.6720±0.1873 | 0.6278±0.1154 | 0.7602±0.0930 |
| DT + LASSO + PCA-40 | 0.6400±0.1020 | 0.5794±0.1791 | 0.5620±0.1869 | 0.5630±0.1641 | 0.6281±0.1116 |
| GBDT + LASSO + PCA-40 | 0.6160±0.0607 | 0.5798±0.1800 | 0.5021±0.0887 | 0.5266±0.0902 | 0.6792±0.0965 |
| LDA + LASSO + PCA-40 | 0.6800±0.1200 | 0.6170±0.2011 | 0.6909±0.2474 | 0.6361±0.1841 | 0.7370±0.1123 |
| Ada + LASSO + PCA-40 | 0.6560±0.0727 | 0.6262±0.1904 | 0.5390±0.1577 | 0.5646±0.1347 | 0.7245±0.1161 |
| RF + LASSO + PCA-50 | 0.6400±0.0283 | 0.6643±0.2161 | 0.4726±0.1187 | 0.5225±0.0598 | 0.6976±0.1338 |
| MLP + LASSO + PCA-50 | 0.6720±0.0335 | 0.6445±0.1433 | 0.6434±0.2107 | 0.6146±0.0879 | 0.7453±0.1447 |
| KNN + LASSO + PCA-50 | 0.6240±0.1081 | 0.6017±0.2282 | 0.4015±0.1533 | 0.4721±0.1672 | 0.6707±0.1447 |
| SVM + LASSO + PCA-50 | 0.6640±0.0607 | 0.6622±0.2274 | 0.5914±0.2548 | 0.5817±0.1366 | 0.7154±0.1427 |
| LR + LASSO + PCA-50 | 0.6640±0.0607 | 0.6211±0.1662 | 0.6532±0.1759 | 0.6162±0.1078 | 0.7455±0.1160 |
| DT + LASSO + PCA-50 | 0.6720±0.1145 | 0.5936±0.1809 | 0.6138±0.2160 | 0.6005±0.1935 | 0.6616±0.1303 |
| GBDT + LASSO + PCA-50 | 0.6080±0.0716 | 0.5611±0.1948 | 0.4344±0.0832 | 0.4870±0.1273 | 0.6606±0.0965 |
| LDA + LASSO + PCA-50 | 0.6320±0.0769 | 0.5781±0.1587 | 0.5812±0.1959 | 0.5640±0.1380 | 0.7276±0.1246 |
| Ada + LASSO + PCA-50 | 0.6080±0.0769 | 0.6037±0.2085 | 0.4526±0.0986 | 0.4956±0.0856 | 0.6906±0.1241 |
| RF + LASSO + PCA-60 | 0.6560±0.0780 | 0.6716±0.2490 | 0.5091±0.1710 | 0.5515±0.1202 | 0.7032±0.1304 |
| MLP + LASSO + PCA-60 | 0.6560±0.0829 | 0.6267±0.1810 | 0.5880±0.2416 | 0.5771±0.1496 | 0.7401±0.1336 |
| KNN + LASSO + PCA-60 | 0.6240±0.1152 | 0.6502±0.2878 | 0.3308±0.2014 | 0.4120±0.2083 | 0.6744±0.1985 |
| SVM + LASSO + PCA-60 | 0.6160±0.0780 | 0.6229±0.2592 | 0.4693±0.1519 | 0.5037±0.1199 | 0.7005±0.1501 |
| LR + LASSO + PCA-60 | 0.6160±0.0358 | 0.5659±0.1047 | 0.6199±0.1159 | 0.5774±0.0350 | 0.7002±0.1031 |
| DT + LASSO + PCA-60 | 0.6400±0.0693 | 0.5960±0.1774 | 0.4909±0.1680 | 0.5243±0.1631 | 0.6222±0.0885 |
| GBDT + LASSO + PCA-60 | 0.6400±0.0490 | 0.6179±0.1741 | 0.5021±0.0887 | 0.5418±0.0836 | 0.6668±0.1196 |
| LDA + LASSO + PCA-60 | 0.6160±0.1043 | 0.5464±0.1447 | 0.6034±0.2275 | 0.5624±0.1569 | 0.6759±0.1149 |
| Ada + LASSO + PCA-60 | 0.6560±0.0669 | 0.6156±0.1584 | 0.5921±0.0865 | 0.5947±0.0909 | 0.7001±0.1170 |
| RF + LASSO + ISOMAP-10 | 0.6880±0.0593 | 0.6772±0.1775 | 0.6045±0.1809 | 0.6153±0.1043 | 0.7518±0.1728 |
| MLP + LASSO + ISOMAP-10 | 0.6640±0.0456 | 0.6154±0.1288 | 0.6353±0.1071 | 0.6154±0.0719 | 0.7398±0.1124 |
| KNN + LASSO + ISOMAP-10 | 0.7200±0.1327 | 0.7464±0.2479 | 0.5344±0.1509 | 0.6211±0.1856 | 0.7722±0.1495 |
| SVM + LASSO + ISOMAP-10 | 0.7200±0.0490 | 0.6783±0.1061 | 0.6975±0.1847 | 0.6732±0.0880 | 0.7594±0.1157 |
| LR + LASSO + ISOMAP-10 | 0.6320±0.0335 | 0.5772±0.1057 | 0.6353±0.1071 | 0.5937±0.0508 | 0.7518±0.1037 |
| DT + LASSO + ISOMAP-10 | 0.6160±0.0727 | 0.5581±0.2106 | 0.5145±0.2298 | 0.5155±0.1767 | 0.6073±0.1006 |
| GBDT + LASSO + ISOMAP-10 | 0.7040±0.1004 | 0.7093±0.2378 | 0.5532±0.1560 | 0.6109±0.1559 | 0.7413±0.1585 |
| LDA + LASSO + ISOMAP-10 | 0.6800±0.0894 | 0.6257±0.1457 | 0.6375±0.2274 | 0.6174±0.1564 | 0.7651±0.1100 |
| Ada + LASSO + ISOMAP-10 | 0.7040±0.1539 | 0.6684±0.2545 | 0.6788±0.2573 | 0.6484±0.2015 | 0.7348±0.1879 |
| RF + LASSO + ISOMAP-20 | 0.6960±0.0727 | 0.6789±0.1824 | 0.6434±0.2107 | 0.6337±0.1207 | 0.7490±0.1574 |
| MLP + LASSO + ISOMAP-20 | 0.6640±0.0456 | 0.5983±0.1036 | 0.6686±0.1664 | 0.6215±0.1045 | 0.7003±0.1266 |
| KNN + LASSO + ISOMAP-20 | 0.6960±0.1187 | 0.6534±0.1806 | 0.5621±0.2230 | 0.5968±0.1912 | 0.7262±0.1410 |
| SVM + LASSO + ISOMAP-20 | 0.6640±0.0669 | 0.6157±0.1586 | 0.6186±0.2346 | 0.5955±0.1454 | 0.7359±0.1433 |
| LR + LASSO + ISOMAP-20 | 0.6480±0.0335 | 0.5744±0.0781 | 0.6686±0.2296 | 0.6017±0.1317 | 0.7417±0.1109 |
| DT + LASSO + ISOMAP-20 | 0.6640±0.1117 | 0.6124±0.2253 | 0.5807±0.2033 | 0.5848±0.1891 | 0.6558±0.1288 |
| GBDT + LASSO + ISOMAP-20 | 0.6640±0.1152 | 0.6404±0.2513 | 0.5699±0.1642 | 0.5888±0.1634 | 0.7414±0.1729 |
| LDA + LASSO + ISOMAP-20 | 0.6720±0.0769 | 0.5963±0.1185 | 0.6699±0.2477 | 0.6174±0.1612 | 0.7294±0.1110 |
| Ada + LASSO + ISOMAP-20 | 0.6720±0.1277 | 0.6433±0.1985 | 0.6156±0.1767 | 0.6123±0.1566 | 0.7328±0.1768 |
| RF + LASSO + ISOMAP-30 | 0.6720±0.0593 | 0.6714±0.1728 | 0.5712±0.2001 | 0.5872±0.1020 | 0.7195±0.1667 |
| MLP + LASSO + ISOMAP-30 | 0.6480±0.0820 | 0.5832±0.1548 | 0.6375±0.2274 | 0.5947±0.1508 | 0.7115±0.1263 |
| KNN + LASSO + ISOMAP-30 | 0.6400±0.0490 | 0.6450±0.1218 | 0.3747±0.1069 | 0.4668±0.0890 | 0.7224±0.1333 |
| SVM + LASSO + ISOMAP-30 | 0.6800±0.0748 | 0.6434±0.1551 | 0.5786±0.1868 | 0.5970±0.1427 | 0.7487±0.1227 |
| LR + LASSO + ISOMAP-30 | 0.6320±0.0657 | 0.5594±0.1074 | 0.6409±0.2576 | 0.5801±0.1437 | 0.7484±0.1159 |
| DT + LASSO + ISOMAP-30 | 0.6320±0.0955 | 0.5800±0.1859 | 0.5651±0.2070 | 0.5502±0.1796 | 0.6247±0.1043 |
| GBDT + LASSO + ISOMAP-30 | 0.6480±0.1110 | 0.6221±0.2617 | 0.5277±0.1606 | 0.5584±0.1709 | 0.7103±0.1662 |
| LDA + LASSO + ISOMAP-30 | 0.6880±0.0912 | 0.6324±0.1351 | 0.6409±0.2576 | 0.6204±0.1655 | 0.7487±0.0959 |
| Ada + LASSO + ISOMAP-30 | 0.6640±0.1374 | 0.6579±0.2409 | 0.6134±0.2078 | 0.6043±0.1573 | 0.7120±0.1563 |
| RF + LASSO + ISOMAP-40 | 0.6960±0.0921 | 0.7168±0.2277 | 0.5803±0.1426 | 0.6154±0.1202 | 0.7905±0.1462 |
| MLP + LASSO + ISOMAP-40 | 0.6880±0.0769 | 0.6492±0.0940 | 0.6634±0.1850 | 0.6397±0.0922 | 0.7633±0.1192 |
| KNN + LASSO + ISOMAP-40 | 0.6400±0.0632 | 0.5933±0.1906 | 0.4269±0.2187 | 0.4785±0.1982 | 0.7149±0.1178 |
| SVM + LASSO + ISOMAP-40 | 0.6800±0.0894 | 0.6545±0.1613 | 0.5645±0.1902 | 0.5928±0.1467 | 0.7924±0.1088 |
| LR + LASSO + ISOMAP-40 | 0.7200±0.0632 | 0.6720±0.0492 | 0.6988±0.1890 | 0.6740±0.0914 | 0.7725±0.1148 |
| DT + LASSO + ISOMAP-40 | 0.6080±0.1481 | 0.4983±0.2210 | 0.6305±0.3117 | 0.5465±0.2655 | 0.6074±0.1546 |
| GBDT + LASSO + ISOMAP-40 | 0.6880±0.0716 | 0.6668±0.1618 | 0.6344±0.0648 | 0.6365±0.0680 | 0.7871±0.1215 |
| LDA + LASSO + ISOMAP-40 | 0.7200±0.0938 | 0.6696±0.0982 | 0.7001±0.2092 | 0.6729±0.1272 | 0.7943±0.1326 |
| Ada + LASSO + ISOMAP-40 | 0.6400±0.0800 | 0.6326±0.1309 | 0.5206±0.2124 | 0.5365±0.1340 | 0.7064±0.0981 |
| RF + LASSO + ISOMAP-50 | 0.6800±0.0566 | 0.7399±0.2483 | 0.5090±0.1181 | 0.5735±0.0677 | 0.7856±0.1543 |
| MLP + LASSO + ISOMAP-50 | 0.6960±0.0727 | 0.6649±0.1593 | 0.6310±0.1627 | 0.6320±0.1202 | 0.7555±0.1306 |
| KNN + LASSO + ISOMAP-50 | 0.6160±0.0607 | 0.6622±0.2496 | 0.3171±0.0874 | 0.4123±0.0818 | 0.7422±0.1282 |
| SVM + LASSO + ISOMAP-50 | 0.6720±0.0867 | 0.6687±0.1769 | 0.5047±0.1607 | 0.5610±0.1428 | 0.7656±0.1152 |
| LR + LASSO + ISOMAP-50 | 0.6560±0.0669 | 0.5908±0.0668 | 0.6199±0.2311 | 0.5907±0.1364 | 0.7466±0.0833 |
| DT + LASSO + ISOMAP-50 | 0.5760±0.1043 | 0.4766±0.1710 | 0.5545±0.2936 | 0.4945±0.2246 | 0.5757±0.1159 |
| GBDT + LASSO + ISOMAP-50 | 0.6720±0.1246 | 0.6576±0.2050 | 0.5934±0.1259 | 0.6087±0.1376 | 0.7864±0.1392 |
| LDA + LASSO + ISOMAP-50 | 0.6640±0.0829 | 0.5938±0.0587 | 0.6379±0.2480 | 0.6011±0.1518 | 0.7622±0.1093 |
| Ada + LASSO + ISOMAP-50 | 0.6560±0.0456 | 0.6661±0.1906 | 0.5571±0.1856 | 0.5691±0.0808 | 0.7198±0.0811 |
| RF + LASSO + ISOMAP-60 | 0.6560±0.0921 | 0.6670±0.2439 | 0.5269±0.1764 | 0.5594±0.1372 | 0.7493±0.1638 |
| MLP + LASSO + ISOMAP-60 | 0.6960±0.0921 | 0.6976±0.2218 | 0.5721±0.1187 | 0.6148±0.1296 | 0.7498±0.1045 |
| KNN + LASSO + ISOMAP-60 | 0.6240±0.0358 | 0.7317±0.2648 | 0.3184±0.1091 | 0.4108±0.0759 | 0.6587±0.0982 |
| SVM + LASSO + ISOMAP-60 | 0.6240±0.0727 | 0.6134±0.1886 | 0.4447±0.1205 | 0.4986±0.1084 | 0.7564±0.0858 |
| LR + LASSO + ISOMAP-60 | 0.6960±0.0537 | 0.6597±0.0812 | 0.6212±0.1823 | 0.6263±0.1048 | 0.7343±0.0612 |
| DT + LASSO + ISOMAP-60 | 0.6160±0.0727 | 0.5542±0.1343 | 0.5718±0.1725 | 0.5506±0.1318 | 0.6132±0.0755 |
| GBDT + LASSO + ISOMAP-60 | 0.6640±0.1345 | 0.6817±0.2614 | 0.5755±0.0275 | 0.6040±0.1191 | 0.7539±0.1590 |
| LDA + LASSO + ISOMAP-60 | 0.6880±0.0438 | 0.6371±0.0931 | 0.6520±0.1603 | 0.6337±0.0941 | 0.7314±0.0315 |
| Ada + LASSO + ISOMAP-60 | 0.6560±0.0358 | 0.6475±0.2031 | 0.5623±0.0818 | 0.5819±0.0403 | 0.6794±0.0892 |
| RF + LASSO + UMAP-10 | 0.7040±0.0829 | 0.6940±0.2097 | 0.6621±0.2226 | 0.6467±0.1252 | 0.7755±0.1557 |
| MLP + LASSO + UMAP-10 | 0.6400±0.0490 | 0.5899±0.1267 | 0.6131±0.1450 | 0.5870±0.0865 | 0.7347±0.1016 |
| KNN + LASSO + UMAP-10 | 0.7040±0.1004 | 0.6927±0.1762 | 0.5610±0.1875 | 0.6105±0.1611 | 0.7678±0.1455 |
| SVM + LASSO + UMAP-10 | 0.6880±0.0593 | 0.6510±0.1301 | 0.6186±0.1733 | 0.6207±0.1104 | 0.7358±0.1355 |
| LR + LASSO + UMAP-10 | 0.6560±0.0456 | 0.6177±0.1524 | 0.5999±0.1463 | 0.5930±0.0864 | 0.7507±0.1003 |
| DT + LASSO + UMAP-10 | 0.6160±0.0537 | 0.5329±0.1237 | 0.5642±0.2170 | 0.5368±0.1708 | 0.6065±0.0750 |
| GBDT + LASSO + UMAP-10 | 0.7200±0.0938 | 0.7232±0.2257 | 0.5699±0.1642 | 0.6278±0.1577 | 0.7695±0.1594 |
| LDA + LASSO + UMAP-10 | 0.6800±0.0693 | 0.6563±0.1738 | 0.5377±0.1406 | 0.5836±0.1319 | 0.7789±0.0980 |
| Ada + LASSO + UMAP-10 | 0.7120±0.1110 | 0.6833±0.2214 | 0.6621±0.2328 | 0.6486±0.1683 | 0.7555±0.1638 |
| RF + LASSO + UMAP-20 | 0.6720±0.1308 | 0.6485±0.2434 | 0.6199±0.2311 | 0.6097±0.1791 | 0.7789±0.1776 |
| MLP + LASSO + UMAP-20 | 0.6240±0.0727 | 0.5667±0.1545 | 0.6075±0.1469 | 0.5749±0.1168 | 0.7264±0.0997 |
| KNN + LASSO + UMAP-20 | 0.7040±0.0780 | 0.7167±0.1988 | 0.5355±0.0794 | 0.6080±0.1122 | 0.7758±0.1671 |
| SVM + LASSO + UMAP-20 | 0.6800±0.0849 | 0.6327±0.1648 | 0.5918±0.1953 | 0.6003±0.1585 | 0.7517±0.1252 |
| LR + LASSO + UMAP-20 | 0.6480±0.0438 | 0.6100±0.1583 | 0.5999±0.1463 | 0.5874±0.0838 | 0.7545±0.0934 |
| DT + LASSO + UMAP-20 | 0.6080±0.1110 | 0.5356±0.2428 | 0.4831±0.2046 | 0.5008±0.2071 | 0.5912±0.1327 |
| GBDT + LASSO + UMAP-20 | 0.6960±0.0963 | 0.6762±0.2272 | 0.5653±0.1350 | 0.6095±0.1543 | 0.7761±0.1729 |
| LDA + LASSO + UMAP-20 | 0.6720±0.0769 | 0.6551±0.1727 | 0.5223±0.1447 | 0.5715±0.1291 | 0.7758±0.1002 |
| Ada + LASSO + UMAP-20 | 0.7360±0.1403 | 0.6959±0.2380 | 0.6720±0.2283 | 0.6719±0.1990 | 0.7752±0.1707 |
| RF + LASSO + UMAP-30 | 0.6320±0.0657 | 0.6048±0.1823 | 0.5499±0.2366 | 0.5431±0.1392 | 0.7326±0.1755 |
| MLP + LASSO + UMAP-30 | 0.6400±0.0748 | 0.5794±0.1350 | 0.6540±0.1462 | 0.6041±0.1003 | 0.7231±0.0979 |
| KNN + LASSO + UMAP-30 | 0.6800±0.1166 | 0.6622±0.2227 | 0.5199±0.2083 | 0.5727±0.1908 | 0.7781±0.1644 |
| SVM + LASSO + UMAP-30 | 0.6560±0.0963 | 0.6083±0.1618 | 0.5585±0.2266 | 0.5640±0.1735 | 0.7512±0.1469 |
| LR + LASSO + UMAP-30 | 0.6800±0.0632 | 0.6358±0.1520 | 0.6353±0.1627 | 0.6225±0.1100 | 0.7679±0.1063 |
| DT + LASSO + UMAP-30 | 0.6480±0.1145 | 0.5689±0.1976 | 0.5816±0.2394 | 0.5679±0.2071 | 0.6384±0.1319 |
| GBDT + LASSO + UMAP-30 | 0.6880±0.0820 | 0.6620±0.2223 | 0.5866±0.1800 | 0.6069±0.1498 | 0.7583±0.1689 |
| LDA + LASSO + UMAP-30 | 0.6560±0.0607 | 0.6309±0.1791 | 0.5223±0.1447 | 0.5582±0.1200 | 0.7746±0.1057 |
| Ada + LASSO + UMAP-30 | 0.6640±0.1315 | 0.6557±0.2597 | 0.5703±0.2332 | 0.5815±0.1851 | 0.7352±0.1790 |
| RF + LASSO + UMAP-40 | 0.7120±0.0769 | 0.6885±0.1390 | 0.6655±0.2096 | 0.6556±0.1077 | 0.7464±0.1607 |
| MLP + LASSO + UMAP-40 | 0.6720±0.0438 | 0.6150±0.1011 | 0.6896±0.1381 | 0.6384±0.0674 | 0.7297±0.1100 |
| KNN + LASSO + UMAP-40 | 0.6640±0.0921 | 0.6377±0.1797 | 0.5188±0.1439 | 0.5652±0.1458 | 0.7396±0.1534 |
| SVM + LASSO + UMAP-40 | 0.6720±0.0912 | 0.6237±0.1474 | 0.5807±0.2446 | 0.5822±0.1758 | 0.7609±0.1421 |
| LR + LASSO + UMAP-40 | 0.6480±0.0522 | 0.6100±0.1583 | 0.5999±0.1463 | 0.5879±0.0860 | 0.7535±0.1076 |
| DT + LASSO + UMAP-40 | 0.6320±0.1110 | 0.5918±0.2382 | 0.5210±0.1981 | 0.5395±0.1795 | 0.6255±0.1292 |
| GBDT + LASSO + UMAP-40 | 0.7440±0.1187 | 0.7184±0.2198 | 0.6340±0.1592 | 0.6711±0.1805 | 0.7633±0.1770 |
| LDA + LASSO + UMAP-40 | 0.6800±0.0748 | 0.6540±0.1619 | 0.5377±0.1406 | 0.5845±0.1323 | 0.7655±0.1085 |
| Ada + LASSO + UMAP-40 | 0.6880±0.1073 | 0.6576±0.1851 | 0.6112±0.2033 | 0.6176±0.1551 | 0.7529±0.1708 |
| RF + LASSO + UMAP-50 | 0.6800±0.1020 | 0.6410±0.1939 | 0.6566±0.1961 | 0.6309±0.1401 | 0.7651±0.1702 |
| MLP + LASSO + UMAP-50 | 0.6720±0.0522 | 0.6184±0.1237 | 0.6874±0.1188 | 0.6389±0.0711 | 0.7306±0.1122 |
| KNN + LASSO + UMAP-50 | 0.7600±0.0894 | 0.7771±0.1281 | 0.6131±0.1450 | 0.6828±0.1336 | 0.7937±0.1814 |
| SVM + LASSO + UMAP-50 | 0.6720±0.0955 | 0.6252±0.1440 | 0.5853±0.2655 | 0.5824±0.1785 | 0.7608±0.1440 |
| LR + LASSO + UMAP-50 | 0.6720±0.0657 | 0.6242±0.1545 | 0.6520±0.1603 | 0.6237±0.1063 | 0.7849±0.1186 |
| DT + LASSO + UMAP-50 | 0.6400±0.1020 | 0.6143±0.2406 | 0.5845±0.2120 | 0.5728±0.1477 | 0.6426±0.1206 |
| GBDT + LASSO + UMAP-50 | 0.6400±0.1166 | 0.6389±0.2576 | 0.5179±0.1224 | 0.5538±0.1310 | 0.7434±0.1591 |
| LDA + LASSO + UMAP-50 | 0.7120±0.0912 | 0.6838±0.1733 | 0.6166±0.2052 | 0.6359±0.1606 | 0.7967±0.1354 |
| Ada + LASSO + UMAP-50 | 0.6880±0.0867 | 0.6601±0.1846 | 0.6112±0.2033 | 0.6158±0.1419 | 0.7444±0.1330 |
| RF + LASSO + UMAP-60 | 0.6560±0.1043 | 0.6239±0.1975 | 0.6066±0.2823 | 0.5817±0.1681 | 0.7352±0.1566 |
| MLP + LASSO + UMAP-60 | 0.6640±0.0537 | 0.6131±0.1406 | 0.6320±0.1425 | 0.6106±0.1026 | 0.7227±0.1110 |
| KNN + LASSO + UMAP-60 | 0.7120±0.0769 | 0.7148±0.1271 | 0.5610±0.1231 | 0.6233±0.1095 | 0.7595±0.1683 |
| SVM + LASSO + UMAP-60 | 0.6800±0.0800 | 0.6394±0.1242 | 0.5785±0.2011 | 0.5932±0.1474 | 0.7507±0.1439 |
| LR + LASSO + UMAP-60 | 0.6160±0.0607 | 0.5774±0.1752 | 0.5577±0.0994 | 0.5523±0.0816 | 0.7338±0.0884 |
| DT + LASSO + UMAP-60 | 0.6000±0.1095 | 0.5167±0.1923 | 0.4709±0.2362 | 0.4872±0.2051 | 0.5815±0.1347 |
| GBDT + LASSO + UMAP-60 | 0.6880±0.0657 | 0.6718±0.2044 | 0.5912±0.1699 | 0.6099±0.1203 | 0.7519±0.1680 |
| LDA + LASSO + UMAP-60 | 0.6800±0.0748 | 0.6540±0.1619 | 0.5377±0.1406 | 0.5845±0.1323 | 0.7631±0.0849 |
| Ada + LASSO + UMAP-60 | 0.6640±0.1152 | 0.6219±0.1898 | 0.6099±0.1816 | 0.6042±0.1528 | 0.7301±0.1716 |
| RF + LASSO + SPECTRAL-10 | 0.7520±0.0867 | 0.7354±0.1863 | 0.6955±0.2203 | 0.6943±0.1437 | 0.7962±0.1700 |
| MLP + LASSO + SPECTRAL-10 | 0.6560±0.0358 | 0.6051±0.1200 | 0.6553±0.1293 | 0.6159±0.0614 | 0.7329±0.1213 |
| KNN + LASSO + SPECTRAL-10 | 0.7120±0.1368 | 0.6717±0.1883 | 0.6123±0.2277 | 0.6339±0.2010 | 0.7702±0.1667 |
| SVM + LASSO + SPECTRAL-10 | 0.6960±0.0876 | 0.6402±0.1639 | 0.6886±0.1904 | 0.6511±0.1389 | 0.7553±0.1357 |
| LR + LASSO + SPECTRAL-10 | 0.6800±0.0693 | 0.6511±0.2048 | 0.6497±0.1081 | 0.6333±0.0908 | 0.7517±0.1110 |
| DT + LASSO + SPECTRAL-10 | 0.6720±0.0769 | 0.6311±0.1628 | 0.5896±0.1581 | 0.5988±0.1244 | 0.6650±0.0869 |
| GBDT + LASSO + SPECTRAL-10 | 0.7280±0.0912 | 0.7395±0.2098 | 0.5888±0.1393 | 0.6448±0.1358 | 0.7728±0.1429 |
| LDA + LASSO + SPECTRAL-10 | 0.6720±0.0769 | 0.6408±0.1526 | 0.5623±0.1916 | 0.5848±0.1356 | 0.7693±0.1100 |
| Ada + LASSO + SPECTRAL-10 | 0.7120±0.0522 | 0.6856±0.1883 | 0.6399±0.2063 | 0.6415±0.1262 | 0.7591±0.1353 |
| RF + LASSO + SPECTRAL-20 | 0.6880±0.0912 | 0.6882±0.2371 | 0.5999±0.1463 | 0.6200±0.1201 | 0.7773±0.1476 |
| MLP + LASSO + SPECTRAL-20 | 0.6960±0.0727 | 0.6487±0.1653 | 0.7109±0.1539 | 0.6626±0.0998 | 0.7681±0.1325 |
| KNN + LASSO + SPECTRAL-20 | 0.6720±0.1035 | 0.6825±0.2476 | 0.4801±0.1113 | 0.5569±0.1467 | 0.7572±0.1879 |
| SVM + LASSO + SPECTRAL-20 | 0.6960±0.0963 | 0.6411±0.1697 | 0.6664±0.1838 | 0.6441±0.1484 | 0.7474±0.1249 |
| LR + LASSO + SPECTRAL-20 | 0.6960±0.0727 | 0.6656±0.2061 | 0.6920±0.1533 | 0.6572±0.0985 | 0.8113±0.1214 |
| DT + LASSO + SPECTRAL-20 | 0.6560±0.1345 | 0.6057±0.2496 | 0.5185±0.1876 | 0.5557±0.2130 | 0.6384±0.1528 |
| GBDT + LASSO + SPECTRAL-20 | 0.7120±0.0867 | 0.7095±0.1816 | 0.5475±0.1035 | 0.6170±0.1334 | 0.7863±0.1548 |
| LDA + LASSO + SPECTRAL-20 | 0.7040±0.0727 | 0.6700±0.1588 | 0.6566±0.1961 | 0.6459±0.1225 | 0.8189±0.1284 |
| Ada + LASSO + SPECTRAL-20 | 0.7040±0.0669 | 0.6884±0.1980 | 0.6088±0.1094 | 0.6344±0.1037 | 0.7952±0.1306 |
| RF + LASSO + SPECTRAL-30 | 0.7040±0.0607 | 0.7175±0.2150 | 0.6020±0.1907 | 0.6245±0.1131 | 0.7648±0.1613 |
| MLP + LASSO + SPECTRAL-30 | 0.6480±0.0912 | 0.6182±0.2027 | 0.6166±0.1101 | 0.6001±0.1027 | 0.7443±0.1266 |
| KNN + LASSO + SPECTRAL-30 | 0.6560±0.0829 | 0.6250±0.1559 | 0.5279±0.1766 | 0.5606±0.1319 | 0.7211±0.1532 |
| SVM + LASSO + SPECTRAL-30 | 0.6720±0.0657 | 0.6611±0.1940 | 0.5610±0.1231 | 0.5911±0.0980 | 0.7325±0.1216 |
| LR + LASSO + SPECTRAL-30 | 0.6880±0.0820 | 0.6363±0.1667 | 0.6720±0.1873 | 0.6403±0.1307 | 0.7765±0.1088 |
| DT + LASSO + SPECTRAL-30 | 0.6480±0.1110 | 0.6005±0.2334 | 0.5453±0.1688 | 0.5635±0.1796 | 0.6381±0.1276 |
| GBDT + LASSO + SPECTRAL-30 | 0.7280±0.0716 | 0.7593±0.2206 | 0.5509±0.0872 | 0.6315±0.1192 | 0.7773±0.1405 |
| LDA + LASSO + SPECTRAL-30 | 0.7040±0.0607 | 0.6619±0.1474 | 0.6707±0.1279 | 0.6555±0.0938 | 0.7953±0.1227 |
| Ada + LASSO + SPECTRAL-30 | 0.6880±0.0769 | 0.6740±0.2099 | 0.5701±0.1667 | 0.6006±0.1304 | 0.7854±0.1384 |
| RF + LASSO + SPECTRAL-40 | 0.7040±0.0607 | 0.7190±0.2065 | 0.6101±0.2192 | 0.6261±0.1097 | 0.7513±0.1739 |
| MLP + LASSO + SPECTRAL-40 | 0.6480±0.0657 | 0.6348±0.1661 | 0.5656±0.1092 | 0.5783±0.0506 | 0.6876±0.1194 |
| KNN + LASSO + SPECTRAL-40 | 0.6320±0.1180 | 0.6246±0.1918 | 0.4615±0.2032 | 0.5083±0.1627 | 0.7103±0.1438 |
| SVM + LASSO + SPECTRAL-40 | 0.6240±0.1081 | 0.6045±0.2460 | 0.5397±0.1733 | 0.5458±0.1504 | 0.7113±0.1363 |
| LR + LASSO + SPECTRAL-40 | 0.6560±0.0921 | 0.6131±0.1901 | 0.6320±0.1425 | 0.6081±0.1224 | 0.6932±0.1132 |
| DT + LASSO + SPECTRAL-40 | 0.6560±0.1152 | 0.6164±0.2313 | 0.5464±0.1852 | 0.5700±0.1805 | 0.6457±0.1344 |
| GBDT + LASSO + SPECTRAL-40 | 0.7280±0.0657 | 0.7619±0.2208 | 0.5532±0.1560 | 0.6269±0.1304 | 0.7815±0.1754 |
| LDA + LASSO + SPECTRAL-40 | 0.6320±0.0593 | 0.5740±0.1397 | 0.6520±0.1034 | 0.6008±0.0813 | 0.7013±0.1066 |
| Ada + LASSO + SPECTRAL-40 | 0.6960±0.0921 | 0.6495±0.1542 | 0.6255±0.1387 | 0.6325±0.1326 | 0.7370±0.1196 |
| RF + LASSO + SPECTRAL-50 | 0.6720±0.1110 | 0.6733±0.2385 | 0.5853±0.2132 | 0.5937±0.1573 | 0.7708±0.1618 |
| MLP + LASSO + SPECTRAL-50 | 0.6720±0.0867 | 0.6267±0.1781 | 0.6523±0.2204 | 0.6170±0.1414 | 0.6798±0.1231 |
| KNN + LASSO + SPECTRAL-50 | 0.6560±0.1252 | 0.6474±0.1832 | 0.5149±0.1686 | 0.5598±0.1545 | 0.7148±0.1520 |
| SVM + LASSO + SPECTRAL-50 | 0.6240±0.1004 | 0.6407±0.2666 | 0.4760±0.2086 | 0.5088±0.1438 | 0.7153±0.1416 |
| LR + LASSO + SPECTRAL-50 | 0.6320±0.0955 | 0.5835±0.2002 | 0.6097±0.1862 | 0.5774±0.1506 | 0.6507±0.0879 |
| DT + LASSO + SPECTRAL-50 | 0.6560±0.1931 | 0.5992±0.2883 | 0.5542±0.2815 | 0.5684±0.2732 | 0.6428±0.2115 |
| GBDT + LASSO + SPECTRAL-50 | 0.7200±0.0938 | 0.7486±0.2504 | 0.5520±0.1604 | 0.6211±0.1546 | 0.7884±0.1503 |
| LDA + LASSO + SPECTRAL-50 | 0.6160±0.0727 | 0.5256±0.1428 | 0.6031±0.2393 | 0.5519±0.1841 | 0.6503±0.1137 |
| Ada + LASSO + SPECTRAL-50 | 0.6400±0.0800 | 0.5909±0.1890 | 0.6075±0.1469 | 0.5861±0.1244 | 0.7265±0.1295 |
| RF + LASSO + SPECTRAL-60 | 0.6560±0.0727 | 0.6857±0.2353 | 0.4969±0.2229 | 0.5356±0.1349 | 0.7214±0.1507 |
| MLP + LASSO + SPECTRAL-60 | 0.6160±0.1284 | 0.5570±0.1954 | 0.5979±0.2081 | 0.5640±0.1734 | 0.6628±0.1277 |
| KNN + LASSO + SPECTRAL-60 | 0.5920±0.0955 | 0.5429±0.1483 | 0.3414±0.1429 | 0.4114±0.1382 | 0.6744±0.1127 |
| SVM + LASSO + SPECTRAL-60 | 0.6240±0.1152 | 0.6339±0.2769 | 0.4504±0.1642 | 0.5029±0.1571 | 0.7032±0.1386 |
| LR + LASSO + SPECTRAL-60 | 0.5840±0.0607 | 0.5090±0.1261 | 0.5731±0.1592 | 0.5336±0.1263 | 0.6386±0.1048 |
| DT + LASSO + SPECTRAL-60 | 0.6560±0.0829 | 0.6414±0.2075 | 0.5247±0.1938 | 0.5550±0.1462 | 0.6488±0.0987 |
| GBDT + LASSO + SPECTRAL-60 | 0.6960±0.0921 | 0.6897±0.2437 | 0.5886±0.1361 | 0.6208±0.1379 | 0.7399±0.1464 |
| LDA + LASSO + SPECTRAL-60 | 0.5680±0.0769 | 0.4808±0.1502 | 0.5497±0.1978 | 0.5090±0.1675 | 0.6089±0.1379 |
| Ada + LASSO + SPECTRAL-60 | 0.7200±0.0894 | 0.6884±0.1980 | 0.6396±0.1063 | 0.6581±0.1320 | 0.7837±0.1383 |
